# Above-twofold quantum super-resolution microscopy enabled by multiple idler passes with entangled biphotons

**DOI:** 10.1126/sciadv.aea9457

**Published:** 2026-07-24

**Authors:** Xin Tong, Zhe He, Yide Zhang, Wenyu Liu, Chien-Ying Huang, Lihong V. Wang

**Affiliations:** Caltech Optical Imaging Laboratory, Andrew and Peggy Cherng Department of Medical Engineering, Department of Electrical Engineering, California Institute of Technology, 1200 E. California Blvd., MC 138-78, Pasadena, CA 91125, USA.

## Abstract

Spatial resolution defines the fundamental limit of how precisely optical imaging systems can distinguish details within an object. Recent advances in quantum imaging have surpassed classical resolution limits by exploiting entanglement. However, experimental demonstrations of quantum enhancement above two times using entangled biphotons have thus far been limited to nonimaging experiments that use replication of the object or nonlinearity—both are impractical and inefficient. Here, without resorting to either, we enhance the classical resolution using entangled photon pairs by either two- or fourfold in two system configurations. While the signal photons traverse the object-containing arm only once, the idler photons traverse the symmetric object-free arm either once or thrice. These experiments demonstrate a scalable route toward quantum-correlated super-resolution imaging, motivate theoretical exploration of the underlying mechanisms, and open opportunities for high-precision, low-illumination microscopy.

## Teaser

Entangled biphotons and multipass idler arm design improve spatial resolution beyond twofold.

## INTRODUCTION

Since the inception of optical microscopy, there has been a sustained drive to resolve finer structures within microscopic objects. The spatial resolution of even a perfect optical imaging system is classically limited by diffraction, which depends on the optical wavelength and the numerical aperture (NA) (*1*). To overcome this constraint, researchers have been striving to design super-resolution imaging systems applicable in both the near field (*2*, *3*) and the far field (*4*, *5*) using deterministic (*6*, *7*) or stochastic (*8*, *9*) processes and implemented either experimentally or computationally (*10*). The emergence of entangled photon sources (*11*, *12*) has catalyzed breakthroughs in quantum sensing (*13*, *14*). The entanglement of *N* photons has been used to enhance parameter estimation precision by up to *N*-fold (*15*–*17*). For example, using an *N*-photon entangled “NOON” state produces interference fringes that are *N* times finer than those obtained with classical light (*18*, *19*), and similar spatial frequency enhancements have been achieved with spontaneous parametric down-conversion (SPDC) sources (*20*). Quantum-enhanced optical phase estimation has demonstrated substantial precision gains across a variety of experiments (*21*, *22*). Further improvements in estimation accuracy have been realized experimentally using nonlinear interactions (*23*) or replications of the object (*24*) and analyzed theoretically (*25*–*28*). In the context of imaging, entanglement has been exploited to achieve *N*-fold resolution enhancement by transmitting entangled biphotons (*N* = 2) through the object (*16*). Even when only one photon of the entangled pair interacts with the object (*29*, *30*), a similar resolution improvement has been observed experimentally. However, achieving a stronger quantum advantage in a linear imaging system that interrogates only a single physical copy of the object—without invoking nonlinear processes or using multiple replicas—has remained out of reach in both metrology and imaging.

Here, we introduce an experimental approach that enhances resolution by up to fourfold using a linear optical imaging system. The system uses an SPDC source for widefield illumination and an electron multiplying charge-coupled device camera (EMCCD) for coincidence detection. Alongside our original single-pass design—which yields a twofold resolution improvement—we now incorporate a triple-pass design to attain an even higher resolution enhancement without increasing the number of entangled parties. The system’s design facilitates seamless transition among three distinct imaging configurations to systematically study the scaling regimes: classical imaging (CI), twofold super-resolution imaging (SR2), and fourfold super-resolution imaging (SR4). In one-dimensional (1D) edge spread function (ESF) measurements, SR2 and SR4 produce approximately twofold and fourfold resolution enhancements over CI, respectively, and in two-dimensional microscopy of a resolution target, SR4 outperforms both CI and SR2 as expected. These experimental findings provide a platform for advancing high-precision quantum imaging, and we invite the community to investigate the underlying mechanisms of these enhancements further.

## RESULTS

### Classical and quantum super-resolution imaging

**Fig. 1.**
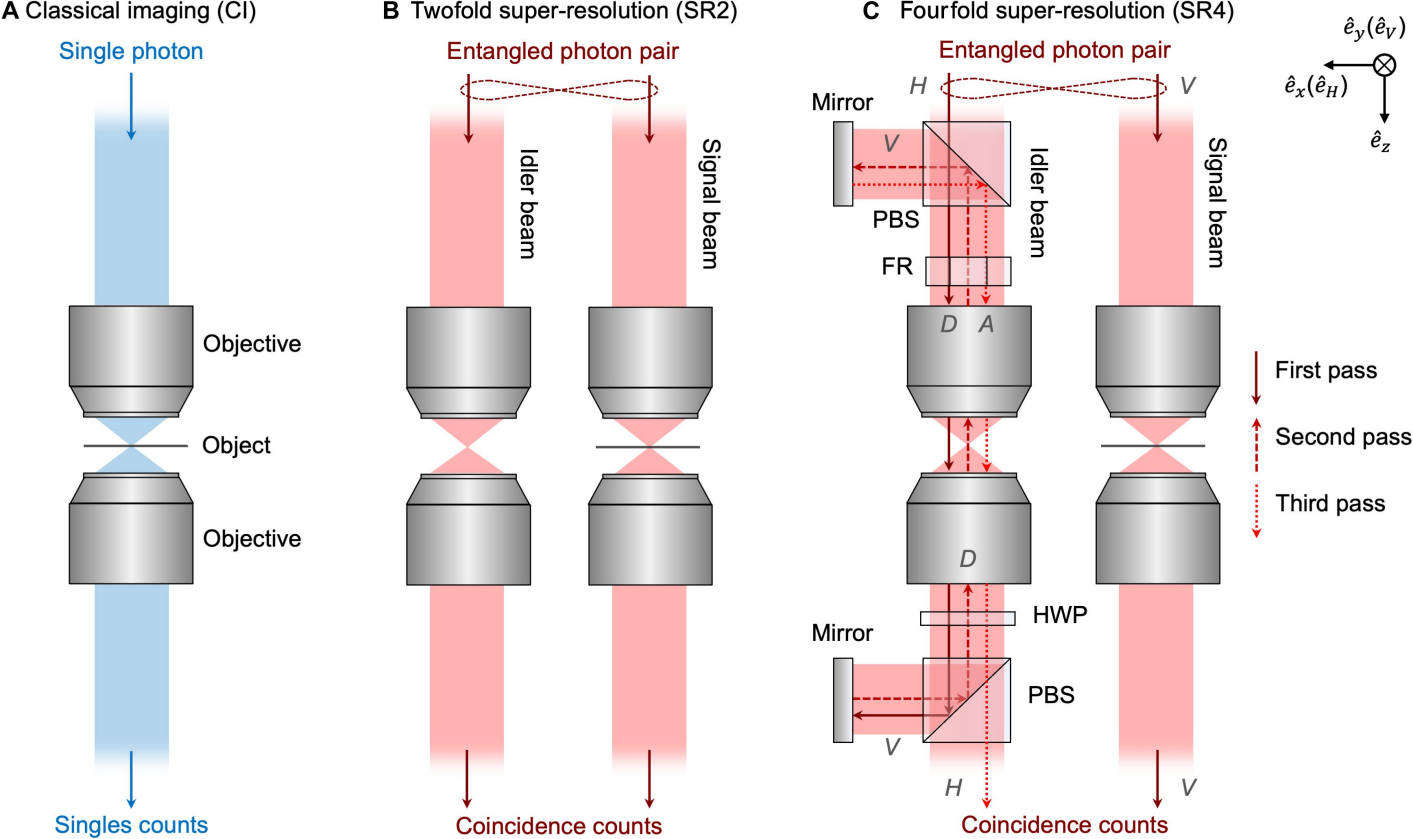
Imaging principles. (**A**) Classical imaging (CI) using single photon counts. (**B**) Twofold super-resolution imaging (SR2) with a single-pass idler arm. (**C**) Fourfold super-resolution imaging (SR4) with a triple-pass idler arm. In the idler arm, the light paths are separated for illustration. PBS, polarizing beam splitter; FR, Faraday rotator; HWP, half-wave plate. *H*, horizontal polarization; *V*, vertical polarization; *D*, diagonal polarization; *A*, antidiagonal polarization.

Figure 1 summarizes the principles of CI, SR2, and SR4. In the CI configuration (Fig. 1A), the single photon counts passing through the object are recorded by the detector to form a CI image. The SR2 configuration (Fig. 1B) attains twofold super resolution using entangled photon pairs. For coincidence detection, an entangled photon pair is split into the idler arm and the signal arm, which transmits the signal photon through the object once. It has been shown that a pair of entangled photons traversing symmetric paths in two arms behaves similar to a single photon with half the original wavelength, which results in a twofold resolution improvement (*30*). Moreover, as shown in Fig. 1C, we reconfigure the single-pass idler arm to enforce theoretically lossless triple passes by incorporating two polarizing beam splitters, a Faraday rotator, and a half-wave plate. This design is inspired by classical multipass microscopy (*31*), in which image contrast, instead of spatial resolution, is enhanced by repeatedly transmitting light through the sample. Our implementation differs in that only the idler arm undergoes three passes, while the signal arm remains single pass. As demonstrated in the following sessions, this design achieved fourfold resolution improvement over CI.

A detailed schematic of the experimental setup can be found in Materials and Methods and fig. S1. We use a β-barium borate (BBO) nonlinear crystal to generate photon pairs, which are simultaneously correlated in position, momentum, and energy through the type-II SPDC process (*32*–*34*). The signal and idler photons are split into two arms by the first prism. The two arms are built symmetrically to ensure symmetric optical wavefronts and magnification ratios. The photon pairs are detected by an EMCCD, and the coincidence counts between the two arms are calculated through a covariance-based algorithm (Materials and Methods and fig. S2) (*30*).

Our experimental setup is designed for versatility, allowing for transitions among the three imaging configurations: CI, SR2, and SR4. The signal arm, by itself, serves as a wide-field microscope, producing CI images. When the fast axis of the half-wave plate is set to 22.5°, the idler beam undergoes a single pass through the objective pair (fig. S3A); under this setting, the coincidences form an SR2 image. When the fast axis of the half-wave plate is set to 67.5°, the idler beam traverses the objective pair thrice (fig. S3B); in this case, the coincidences yield an SR4 image.

### Edge-spread quantification of spatial resolution

**Fig. 2.**
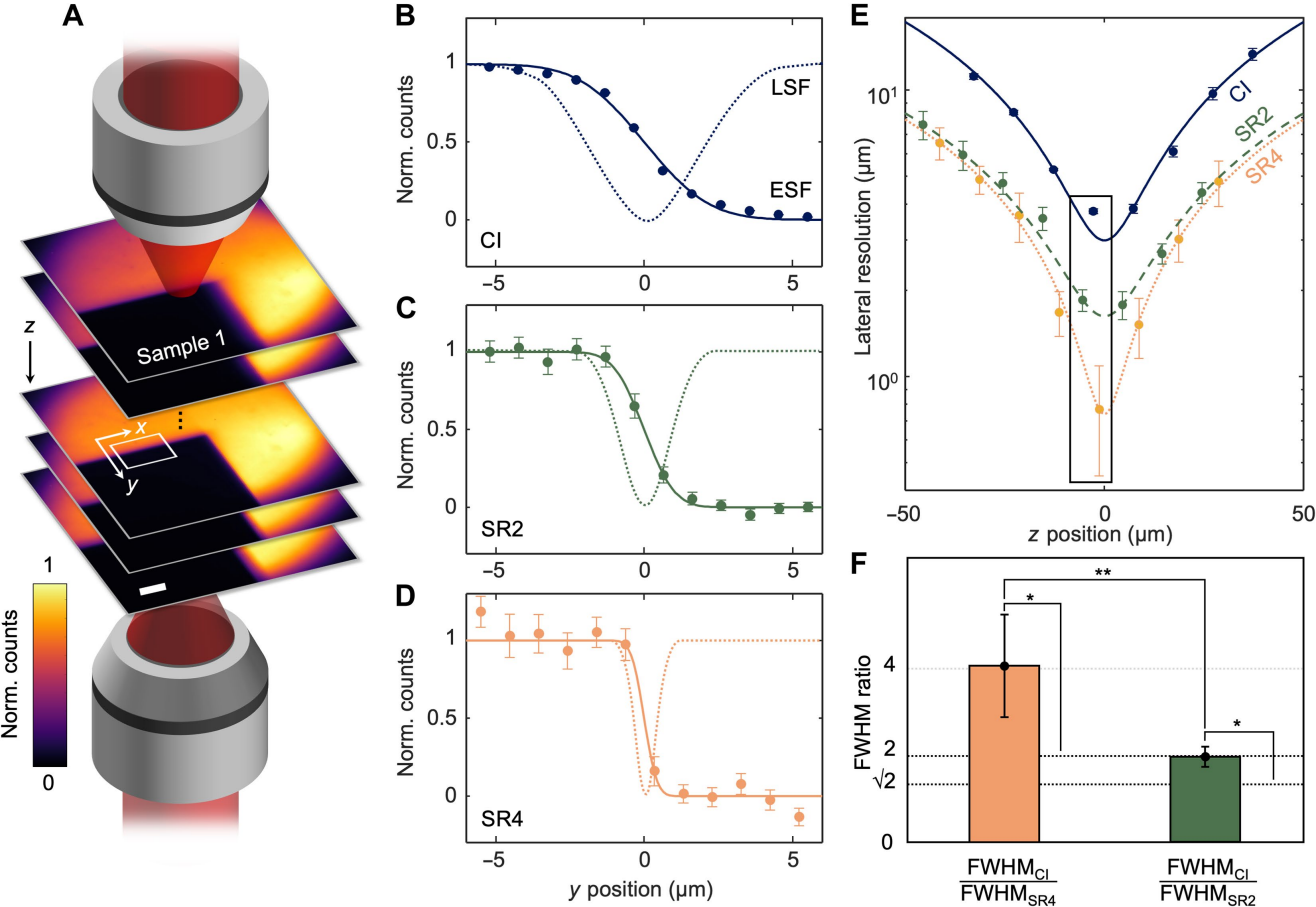
Quantification of spatial resolutions. (**A**) Schematic of the setup for resolution quantification. The USAF target is placed between the objectives, and resolution measurement is repeated along different axial (*z*) positions. (**B** to **D**) Raw and fitted ESFs and LSFs of (B) CI, (**C**) SR2, and (D) SR4 measured near the foci. The raw ESFs are plotted as the means ± SEs of each mean (*n* = 30). Norm., normalized. (**E**) Lateral resolution along *y* versus *z* (optical axis) for CI, SR2, and SR4. Each dot represents the means ± SEM (*n* = 30), and the solid lines denote fits. The three curves are shifted to align the fitted foci where we set *z* = 0. The black box marks the points in (B) to (D). (**F**) FWHM ratios between CI and SR2 or SR4 measured at the *z* positions enclosed by the black box in (E). The *P* values are obtained from one-sided *t*-tests (*n* = 30). *P<0.05 and **P<0.01.

We next quantify the spatial resolutions of the CI, SR2, and SR4 configurations using an edge of a US Air Force (USAF) 1951 resolution target. As shown in Fig. 2A, we select a region of interest along the edge and average the *y* profiles to obtain the raw ESFs. We then fit the ESFs using an error function at different *z* positions varied with a step size of 10 μm. Subsequently, differentiating the fitted ESFs yields the line spread functions (LSFs) whose full widths at half maximum (FWHMs) represent the spatial resolutions as detailed in Fig. 2 (B to D) and Materials and Methods.

Figure 2E shows the *y* lateral resolutions. The curves are fitted in accordance with the Gaussian beam width and shifted slightly (much less than the step size) to align the foci. The minima of the fitted CI, SR2, and SR4 resolution curves, representing the focal spatial resolutions, are 2.98 ± 0.42 μm, 1.52 ± 0.28 μm, and 0.74 ± 0.22 μm, respectively. Relative to CI, SR2 and SR4 enhance the resolution by factors of 1.84 ± 0.41 and 4.03 ± 1.32, respectively. To test if the SR4 resolution is statistically finer than SR2, we estimate the SEs from fitting and perform the one-tailed *t* test against the null hypothesis that the mean values of the FWHM_CI_/FWHM_SR4_ are smaller than those from FWHM_CI_/FWHM_SR2_. We also perform one-tailed *t* tests comparing the measured ratio FWHM_CI_/FWHM_SR2_ with $\sqrt{2}$ and FWHM_CI_/FWHM_SR4_ with 2 (*35*). All corresponding *P* values are below 0.05 (Fig. 2F), indicating the rejection of the null hypotheses with confidence levels above 95%. Similar to the *y* resolution, the *x* resolution exhibits similar proportional enhancements (fig. S4). As an alternative resolution metric, the modulation transfer function (MTF) is included in fig. S5.

To achieve statistical significance, we repeat the measurements on three other samples, as summarized in fig. S6. Again, the average resolution ratios of FWHM_CI_/FWHM_SR2_ and FWHM_CI_/FWHM_SR4_ from each object are around 2 and 4, respectively, and the corresponding *P* values are mostly less than 0.05. We also perform pooled statistical tests including all four samples, and the corresponding *P* values are all less than 0.001.

### Two-dimensional imaging of a USAF target

**Fig. 3.**
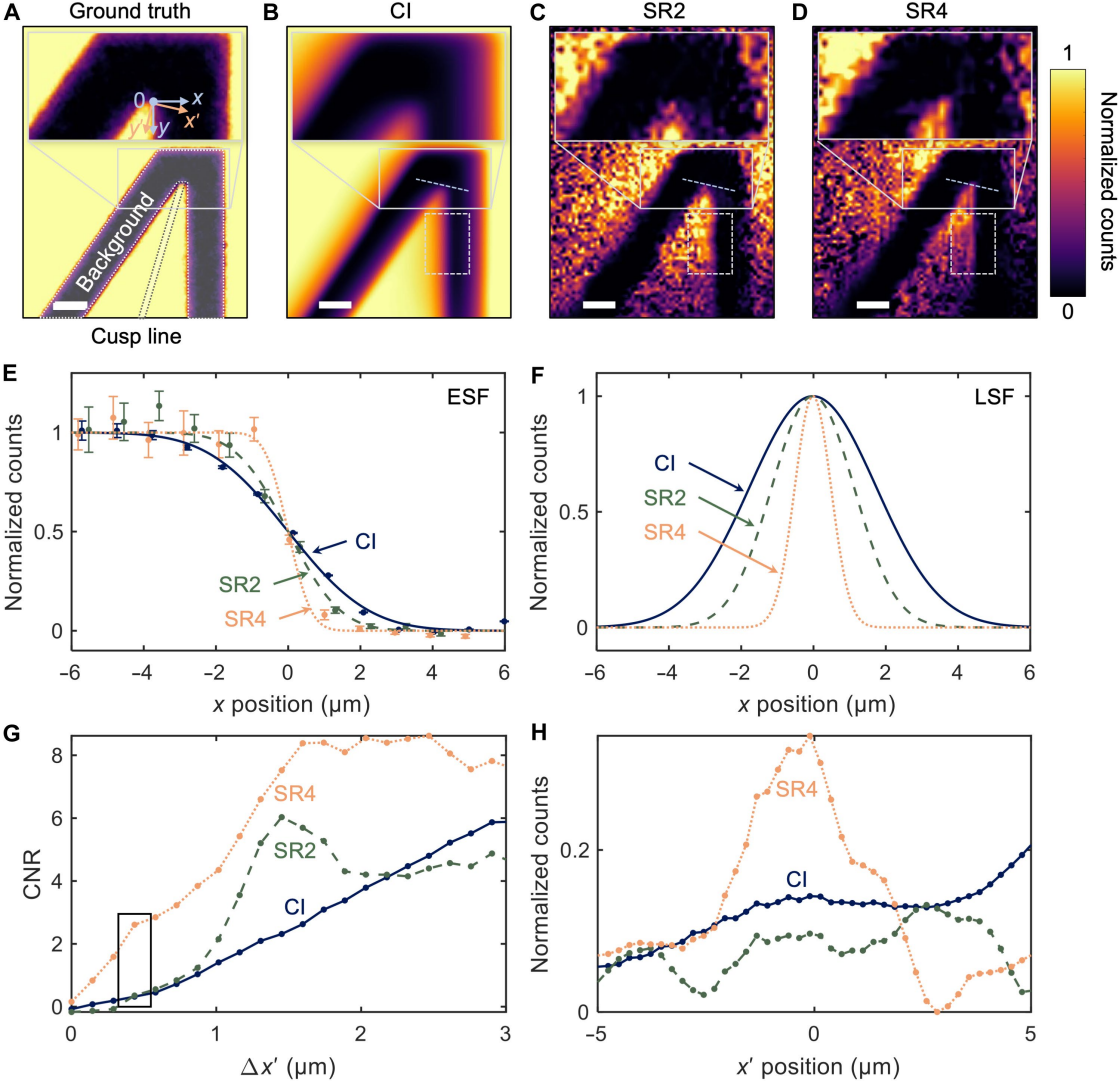
Images of a USAF resolution target. (**A**) Ground-truth (GT), (**B**) CI, (**C**) SR2, and (**D**) SR4 images of the number 4 in group 3 of a USAF 1951 resolution target. The GT image was acquired using white-light optical microscopy under ×40 magnification (resolution: 0.50 μm). The close-up insets of the same region of interest from GT, CI, SR2, and SR4 are compared. The background and the cusp line for CNR computation are marked by dotted contours in (A). Scale bars, 10 μm. (**E** and **F**) Raw and fitted ESFs (E) and the corresponding fitted LSFs (F) of CI, SR2, and SR4 of the edge marked by dashed boxes in (A) to (D). In (E), each dot represents the means ± SEM (*n* = 13), and the lines denote fits. (**G**) CNRs of the $x^{\prime }$ profiles versus $\Delta x^{\prime }$ near the cusp, where the horizontal dashed line indicates CNR=2. The black solid box indicates the position where the SR4 curve initially surpasses 2. (**H**) Normalized CI, SR2, and SR4 counts along the dotted lines in (B) to (D) [and as marked by the black solid box in (G)].

Our microscope allows direct comparison of the two-dimensional images of the same structure across the three configurations. Given the field of view, we image the number “4” in group 3 of the USAF 1951 resolution target (i.e., sample 4 in fig. S6G). To acquire a ground-truth image (Fig. 3A), we first capture the object using a commercial optical microscope with white light at higher magnification (×40). We then image the same region using our setup under the CI, SR2, and SR4 configurations as shown in Fig. 3 (B to D). To facilitate a robust comparison, we register the ground-truth image with the classical image via similarity translation, leading to alignment of all four images in a shared coordinate system. The origins of the coordinates are determined by the edges along the *x* and *y* axes in the ground-truth image.

To quantitatively demonstrate that SR4 can resolve finer than SR2 and CI, we designate two regions of interest within each image. The first region of interest is marked as the dashed boxes in Fig. 3 (A to D), which corresponds to the edge along the vertical stroke of the number “4.” We then fit the raw ESFs (Fig. 3E), and the resolution ratios of SR2/CI and SR4/CI are 1.54 ± 0.51 and 3.50 ± 1.20, respectively.

The second region of interest corresponds to the cusp of the number 4, which is displayed as the insets in Fig. 3 (A to D). We proceed to compute the contrast-to-noise ratios (CNRs) along the rotated $x^{\prime }$ axis perpendicular to the “cusp line” at varying $y^{\prime }$ positions inside the region using the background and signal masks shown in Fig. 3A. The resultant CNRs are plotted in Fig. 3G (see Materials and Methods and fig. S7 for details). SR4 shows a steeper ascent of the CNR curve, resolving the cusp better than SR2 and CI. CNR = 2 indicated by the horizontal dashed line in Fig. 3G means that the signal of interest can be distinguished from the background at approximately a 98% significance level according to the *Z* test. Pinpointing the $y^{\prime }$ position where the SR4 curve initially surpasses 2, we chart the $x^{\prime }$ profiles for all three images in Fig. 3H. A discernible peak proximate to $x^{\prime }=0$ is solely evident in the SR4 profile, revealing the cusp. Note that the CNRs are computed along the 1D $x^{\prime }$ line immediately below the cusp to reveal the resolution and visibility. In contrast, CI, due to its lower noise, shows greater signal-to-noise ratios (SNRs) than SR2 and SR4 over a large region of interest away from the cusp (fig. S7), where spatial resolution becomes less important.

## DISCUSSION

In this work, we report an experimental configuration that achieves up to fourfold quantum resolution enhancement in a linear imaging system using entangled biphotons. In quantum imaging, the localization precision of a photon position *X* can be modeled as a Gaussian random variable $X∼N\left(\mu ,\sigma_{\text{CI}}^{2}\right)$, where the localization accuracy of the estimated mean $\hat{\mu }$ is quantified by the SEM, $\text{SEM}\left(\hat{\mu }\right)$. $\text{SEM}\left(\hat{\mu }\right)$ can be improved by increasing the photon number in two distinct ways. On the one hand (fig. S8C), sending photons that are entangled in $N_{\text{EP}}$ parties improves resolution by reducing $\sigma_{\text{CI}}$ by a factor $f_{\text{EP}}\left(N_{\text{EP}}\right)$ (fig. S8D).

$$\sigma_{\text{EP}}\left(\hat{\mu }\right)=\frac{\sigma_{\text{CI}}}{f_{\text{EP}}\left(N_{\text{EP}}\right)}$$ (1)

On the other hand (fig. S8A), sending $N_{\text{IR}}$ independent repetitions to the detector decreases the estimation error by a factor $f_{\text{IR}}\left(N_{\text{IR}}\right)$ (fig. S8B) by reducing noise; however, these repetitions do not narrow the spread of the original distribution (i.e., the FWHM $\Delta r_{\text{CI}}$ or SD $\sigma_{\text{CI}}$) directly. Combining the two effects yields

$$\text{SEM}\left(\hat{\mu }\right)=\frac{\sigma_{\text{EP}}\left(\hat{\mu }\right)}{f_{\text{IR}}\left(N_{\text{IR}}\right)}$$ (2)

In metrology, scaling improvements associated with $f_{\text{IR}}\left(N_{\text{IR}}\right)\approx N_{\text{IR}}$ have been demonstrated using entanglement or squeezing (*21*, *22*, *36*, *37*). In imaging, achieving $\sqrt{N_{\text{IR}}}$ scaling requires target sparsity (*8*, *9*), while enhancements through $f_{\text{EP}}\left(N_{\text{EP}}\right)$ provide a more efficient alternative. In our SR2 and SR4 configurations, with $N_{\text{EP}}=2$, we experimentally achieve $f_{\text{EP}}\left(2\right)\approx 2$ and $f_{\text{EP}}\left(2\right)\approx 4$, respectively, demonstrating twofold and fourfold resolution enhancements driven by quantum correlations.

Two experimental limitations exist in our study. First, our experimentally achieved best CI resolution of 2.98 μm deviates from the diffraction limit expected from the nominal NA (NA = 0.4) of the objective. This discrepancy is likely attributed to underfilling of the objective aperture, as examined below. To begin with, we rule out significant image distortion caused by the 810-nm SPDC beam by comparing the classical resolution with that obtained using a coherent 532-nm classical laser beam. As shown in fig. S9, the two classical resolutions scale with the wavelengths under similar NAs. We then experimentally measure the effective NA of our setup at three locations along the optical path (fig. S10). Using the beam FWHMs to estimate the actual NA yields a diffraction limit consistent with our experimentally observed classical resolution, as detailed in note S2 and table S1.

The second limitation arises from the relatively high noise levels due to the low photon counts in the coincidence images. In Fig. 3, SR4 exhibits lower SNR than SR2, and both do worse than CI (fig. S7). In the FWHM and MTF analyses, the mean resolution enhancement factors—1.84 ± 0.41 and 4.03 ± 1.32 for the SR2 and SR4, respectively—suffer notable uncertainties. Higher SNR will be needed to tighten the confidence intervals for a more precise determination of the exact enhancement. The reductions in SNR are attributed to the loss of coincidence counts, as reflected in the intensity difference between the single-pass and triple-pass idler beams (fig. S11). This loss is primarily caused by optical imperfections in the idler arm, including nonideal objective coatings and the limited aperture of the Faraday rotator. The EMCCD’s relatively low quantum efficiency at 810 nm further suppresses the SNR compared with our earlier 532-nm implementation (*30*). These losses stem from practical rather than fundamental limitations; improvements in optical coatings, component selection, and alignment precision could substantially enhance the SNR in future designs. Moreover, as shown in Eq. 2, resolution and noise affect localization precision differently. Therefore, CNR is introduced to distinguish resolution from SNR (Materials and Methods). CNR, commonly used in imaging (*38*), determines image quality by quantifying the distinguishability between regions of interest and background noise. In Fig. 3, SR4 exhibits higher CNRs (i.e., better visibility) than SR2 and CI near the cusp of the object (Fig. 3G). To further reduce the influence of low SNR on our measurements, we choose the cusp of number 4 as the imaging target. The cusp has a fine feature size comparable to the resolution limit, and its gradual tapering in shape enables a stable and robust CNR-based assessment of resolution. Future improvement could derive from using more acquisition frames (fig. S12) using more powerful or massively parallel entangled photon sources (*39*), higher transmittance optics, larger-aperture Faraday rotators, and more efficient detectors (*40*). Any post-processing techniques to enhance the CI resolution can also be adopted to quantum imaging for further enhancement. With these improvements, we expect to image more complex targets, including graded transmission and biological objects.

Several alternative reasons have been explored to explain the observed resolution enhancement in SR2 and SR4. First, one might suspect that the resolution is enhanced in one dimension at the expense of the other due to changes in the beam profile through triple passes. However, we have consistently observed resolution enhancement in both the *x* and *y* axes, as shown in fig. S4. Second, one might argue that the idler arm effectively changes magnification through triple-pass coincidence detection in SR4. From the images shown in Fig. 3, the same feature has similar sizes in the CI, SR2, and SR4 images. Third, one might suspect that the observed resolution enhancement could arise from classical or thermal-like sources, which, through second-order intensity correlations, can improve resolution by at most a factor of $\sqrt{2}$ (*41*, *42*). In contrast, in Fig. 2, the statistical tests show that the SR2 resolution-enhancement factor exceeds $\sqrt{2}$ and that SR4 provides an even greater enhancement, surpassing both two times and the SR2 performance. Last, one might suspect that the coincidence detection effectively enlarges the detection NA. As discussed above, the underfilling of the objective aperture suggests headroom for additional resolution gains. Our theory indicates that quantum imaging with disrupted momentum correlation may reduce to an equivalent of classical confocal microscopy (*43*). In that case, a $\sqrt{2}$-fold resolution enhancement is expected when the signal and idler arms share the same NA, and a larger improvement could occur if the idler photons occupy a higher NA. In our setup, the Faraday rotator has a 5-mm aperture, corresponding to an effective NA of 0.28. Given that the measured signal-arm NA is underfilled at ∼0.14 (table S1), the idler arm could at most double the effective NA through multiple passes, yielding a theoretical resolution enhancement of roughly 2 times, which remains statistically smaller than the SR4 enhancement factor observed experimentally. In our system, this potential NA change cannot be directly observed at the EMCCD plane because the idler beam is two orders of magnitude larger than the diffraction spot; it could be quantified through the diffraction size by imaging an idler arm pinhole placed only in the third pass, which deviates substantially from our current configuration. To the contrary, as shown in fig. S13, the correlation peak broadens by ∼20% after the triple passes. This broadening suggests that the effective NA in the idler arm has actually become smaller rather than larger after the triple passes because the peak width reflects the diffraction size determined by the NA. Therefore, none of these postulates could explain the observed resolution enhancement.

Beyond the investigations above, we sought to explain the observed resolution enhancement using the theoretical framework inspired by (*44*) and customized for SR2 and SR4 in our work (notes S3 and S4). In our theory, in the *n*-pass idler arm, the photon detected in coincidence with the signal photon is assumed to accumulate transverse phase from each pass. Since coincidence detection relies on the sum of the transverse phases from both photons, the (*n* + 1)-fold SR resolution is

$$\Delta r_{\text{SR}}=\frac{1}{1+n}\Delta r_{\text{CI}}=\frac{\lambda }{2\left(1+n\right)\;\text{NA}}$$ (3)

where *n* = 1 or 3 and λ is the center wavelength of the SPDC photons. Our theory provides a consistent account of the experimental observations.

In nonimaging metrology, higher-order precision scaling has been theoretically pursued through nonlinear interactions (e.g., Kerr effects) or by replicating the target. Two experimental approaches have demonstrated this enhanced scaling but differ fundamentally from our present work. Napolitano *et al.* (*23*) use nonlinear light-matter interaction, while our SR4 uses a linear imaging system. Yin *et al.* (*24*) replicate the object multiple times in each arm of a Mach-Zehnder interferometer to perturb both conjugate observables (i.e., displacement and momentum) simultaneously but in opposite sequences, whereas our SR4 uses a single instance of the object in one arm and transmits the signal photons only once through the object.

To conclude, by directing idler photons through the objective pair in the idler arm three times and correlating them with the signal photons, we have enhanced the classical spatial resolution using a linear imaging system by a factor of four. This work presents several unexpected findings. For example, the paired pixels are sensitive to the phases from the two arms despite that the two photons do not overlap with each other once departing from the source, and each pixel is sensitive to intensity only. In addition, in our theory, symmetry between the two arms is important to ensure symmetric wavefronts and magnification ratios. Harnessing the distinct advantages of quantum entanglement in a readily scalable manner, we aim to stimulate similar investigations.

## MATERIALS AND METHODS

### Experimental setup

In our system, a BBO crystal [5 mm by 5 mm by 2.0 mm; NCBBO5200-405(II)-BL, Newlight Photonics] is cut for type-II SPDC. The pump is a 405-nm continuous-wave laser (LM-405-PLR-40-4 K, Coherent) with an output power of 40 mW. A Glan-Laser polarizer (GLB10-A, Thorlabs) and a half-wave plate (WPA03-H-405, Newlight Photonics) are used to adjust the polarization angle of the pump laser beam to be horizontally polarized. The pump laser beam then passes through the BBO crystal and generates SPDC photons. A bandpass filter with a center wavelength of 810 nm and a bandwidth of 30 nm (NBF810-30, Newlight Photonics) is used to block the pump beam. The generated SPDC photon pairs propagate through an $f_{0}=50$-mm lens to the Fourier plane, i.e., the source Fourier plane (**P**_0_), and are spatially separated using a knife-edge right-angle prism mirror (MRAK25-P01, Thorlabs). Two polarizing beam splitters (PBS252, Thorlabs), two mirrors, and a Faraday rotator (I780R5, Thorlabs) are used for the triple-pass configuration. The separated signal and idler photons propagate to the object plane ($P_{1,o}$) and the virtual-object plane ($P_{2,o}$), respectively, by two identical 4f imaging systems comprising an $f_{1}=180$-mm lens and an $f_{2}=9$-mm objective (LI-20X, Newport). The sample is placed on the object plane. The object or virtual-object plane and the intermediate plane are conjugated through the other two identical 4f imaging systems, each of which consists of an identical set of $f_{2}=9$-mm objectives and $f_{1}=180$-mm lenses and another right-angle prism mirror. Each objective is followed by a half-wave plate (WPA03-H-810, Newlight Photonics). By rotating the half-wave plate about the optical axis in the idler arm, the system can switch between the SR2 and SR4. The intermediate plane and the detection plane ($P_{\text{det}}$) of an EMCCD camera (iXon Ultra 888, Andor) are conjugated through a 4f system consisting of $f_{3}=300$-mm and $f_{4}=200$-mm lenses. Another band-pass filter (NBF810-30, Newlight Photonics) is placed in front of the EMCCD camera to block unwanted stray light. The EMCCD is operated at −65°C with a horizontal pixel shift readout rate of 10 MHz, a vertical pixel shift speed of 1.13 s, and an electron multiplier gain of 1000. In our experiments, the photon flux is ∼0.4 to 0.5 per pixel per frame in both the signal arm and the single-pass idler arm; however, it is reduced to ∼0.1 in the triple-pass idler arm, primarily due to the cumulative optical loss. Therefore, the coincidence rate is less than 0.05. The whole setup is covered by a light-shielding box.

### Data acquisition and processing

A laboratory-written LabVIEW (National Instruments) program using the library from the Andor software development kit is used to control the EMCCD for data acquisition. The imaging data are saved as 16-bit Flexible Image Transport System (FITS) files, with each file containing 100 frames. The FITS files are imported into MATLAB (MathWorks) and processed with laboratory-written scripts. The EMCCD frames are extracted from the files and are used to calculate the coincidence intensity.

### Coincidence estimation algorithm

We have developed a covariance algorithm to efficiently estimate the coincidence intensity of signal and idler photons using an EMCCD camera (*30*). The signal and idler photons are detected by the left and right regions of the camera, respectively, viewed along the optical axis. The distributions of entangled photon pairs in both regions are symmetric about a center point due to their momentum anticorrelation in the far field of the crystal; therefore, the signal and idler images can be inversely registered pixel by pixel according to the symmetric center. The total intensities of each pair of inversely registered pixels in the signal and idler images are given by

$$I_{1}\left(i_{t}\right)=I_{1}^{\text{coin}}\left(i_{t}\right)+I_{1}^{\text{uncorr}}\left(i_{t}\right)$$ (4)

$$I_{2}\left(i_{t}\right)=I_{2}^{\text{coin}}\left(i_{t}\right)+I_{2}^{\text{uncorr}}\left(i_{t}\right)$$ (5)

where $i_{t}$ denotes the frame index representing a time point, $I^{\text{coin}}$ the reading from the SPDC beam responsible for coincidence, and $I^{\text{uncorr}}$ the reading from other sources (e.g., readout noise) that are uncorrelated with $I^{\text{coin}}$. We use the mean value of coincidence intensity $\bar{I^{\text{coin}}}$ to estimate the intensity correlation $G^{\left(2\right)}$. The mean coincidence intensity is estimated by the covariance between $I_{1}$ and $I_{2}$ defined by

$$\bar{I_{1}^{\text{coin}}}\approx {\text{Cov}}_{t}\left(I_{1},I_{2}\right)=\frac{1}{N_{t}}\sum_{i_{t}}^{N_{t}}\left[I_{1}\left(i_{t}\right)-\bar{I_{1}}\right]\left[I_{2}\left(i_{t}\right)-\bar{I_{2}}\right]$$ (6)

where *N*_t_ is the number of frames. More details of the coincidence estimation algorithm can be found in note S1 and fig. S2.

### Image postprocessing

Upon acquiring the images using the coincidence estimation algorithm, we first map the image intensities to the scale of [0, 1]. Denoting *I* as the image intensity, we normalize using

$$I_{\text{norm}}=\frac{I-I_{\text{min}}}{I_{\text{max}}-I_{\text{min}}}$$ (7)

where *I*_max_ and *I*_min_ are the maximum and minimum values of *I*. The normalized images are then denoised using a block-matching and 3D filtering algorithm (*45*). Representative images before and after denoising are compared in fig. S14.

### SNR and CNR estimation

SNR calculation requires defining signal and background regions. In SNR calculation, we mask the bright regions in the number 4 as the signal (*s*_SNR_) (the black dotted lines in fig. S7). The SNR is therefore computed as

$$\text{SNR}=\frac{\bar{I_{s_{\text{SNR}}}}}{\sigma_{s_{\text{SNR}}}}$$ (8)

which directly reflects the overall image quality.

Other than SNR, we also compute CNRs along the $x^{\prime }$ axis near the cusp at varying $y^{\prime }$ positions in the USAF 4 image. In CNR calculation, we define the cusp line as the middle line between the two arms in 4 as marked by the blue dashed line in fig. S7A. $x^{\prime }$ and $y^{\prime }$ are defined as the axes perpendicular and parallel to the cusp line, respectively. CNR can be computed as a function of $y^{\prime }$ along the 1D $x^{\prime }$ line, which is perpendicular to the cusp line. For each profile, the background region (*b*_CNR_) is defined as the intersection of the line and the (*b*_CNR_) mask, and the signal region (*s*_CNR_) is defined as the intersection of the line and the cusp line. The CNR at $y^{\prime }$ is therefore computed as

$$\text{CNR}\left(y^{\prime }\right)=\frac{\left|I_{s_{\text{CNR}}}-\bar{I_{b_{\text{CNR}}}}\right|}{\sigma_{b_{\text{CNR}}}}$$ (9)

### Quantification of spatial resolution

To quantify the spatial resolution of our system, we extract the intensities along a line perpendicular to an edge in the USAF resolution target and fit them to an ESF using an error function centered at $x_{0}$, i.e.,

$$\text{ESF}\left(x\right)=a\cdot \text{erf}\left[\left(x-x_{0}\right)/w\right]+b$$ (10)

where a and b are coefficients and $\hat{W}$ is the waist radius of the beam. A Gaussian LSF is obtained by taking the derivative of the ESF, i.e.,

$$\text{LSF}\left(x\right)=d\text{ESF}\left(x\right)/\mathrm{dx}=2a\text{exp}\left[-{\left(x-x_{0}\right)}^{2}/w^{2}\right]/\left(w\sqrt{\pi }\right)$$ (11)

The resolution is estimated by the FWHM of the LSF, i.e., $R=2\sqrt{\text{ln}\left(2\right)}w$.

To estimate the SEs from the fitted ESFs, we acquire the 95% confidence interval of the fitted parameter w, which is denoted as $\left[w_{\text{sub}},w_{\text{sup}}\right]$. From the confidence interval, the SEs of w are estimated as ${\text{se}}_{w}=\left(w_{\text{sup}}-w_{\text{sub}}\right)/3.92$ according to the *z* test. From $R=2\sqrt{\text{ln}\left(2\right)}w$, the SEs of the FWHM of the LSFs are estimated as ${\text{se}}_{R}=\sqrt{\text{ln}\left(2\right)}\left(w_{\text{sup}}-w_{\text{sub}}\right)/1.96$.

## Supplementary Materials

### This PDF file includes:

Supplementary Notes S1 to S5 Figs. S1 to S17 Table S1 References

## References

[1] A. Lipson, S. Lipson, H. Lipson, *Optical Physics* (Cambridge Univ. Press, 2012).

[2] E. Betzig, J. Trautman, Near-field optics: Microscopy, spectroscopy, and surface modification beyond the diffraction limit. Science 257, 189–195 (1992). 17794749 10.1126/science.257.5067.189

[3] X. Zhang, Z. Liu, Superlenses to overcome the diffraction limit. Nat. Mater. 7, 435–441 (2008). 18497850 10.1038/nmat2141

[4] M. Minsky, Memoir on inventing the confocal scanning microscope. Scanning 10, 128–138 (1988).

[5] S. Hell, E. Stelzer, Properties of a 4Pi confocal fluorescence microscope. J. Opt. Soc. Am. A 9, 2159–2166 (1992).

[6] S. Hell, J. Wichmann, Breaking the diffraction resolution limit by stimulated emission: Stimulated-emission-depletion fluorescence microscopy. Opt. Lett. 19, 780–782 (1994). 19844443 10.1364/ol.19.000780

[7] M. Gustafsson, Nonlinear structured-illumination microscopy: Wide-field fluorescence imaging with theoretically unlimited resolution. Proc. Natl. Acad. Sci. U.S.A. 102, 13081–13086 (2005). 16141335 10.1073/pnas.0406877102 PMC1201569

[8] E. Betzig, G. Patterson, R. Sougrat, O. Lindwasser, S. Olenych, J. Bonifacino, M. Davidson, J. Lippincott-Schwartz, H. Hess, Imaging intracellular fluorescent proteins at nanometer resolution. Science 313, 1642–1645 (2006). 16902090 10.1126/science.1127344

[9] M. Rust, M. Bates, X. Zhuang, Sub-diffraction-limit imaging by stochastic optical reconstruction microscopy (STORM). Nat. Methods 3, 793–796 (2006). 16896339 10.1038/nmeth929 PMC2700296

[10] C. Ledig, L. Theis, F. Huszár, J. Caballero, A. Cunningham, A. Acosta, A. Aitken, A. Tejani, J. Totz, Z. Wang, W. Shi, Photo-realistic single image super-resolution using a generative adversarial network, in *Proceedings of the IEEE Conference on Computer Vision and Pattern Recognition (CVPR)* (IEEE, 2017), pp. 4681–4690.

[11] P.-A. Moreau, E. Toninelli, T. Gregory, M. Padgett, Imaging with quantum states of light. Nat. Rev. Phys. 1, 367–380 (2019).

[12] R. Horodecki, P. Horodecki, M. Horodecki, K. Horodecki, Quantum entanglement. Rev. Mod. Phys. 81, 865–942 (2009).

[13] V. Giovannetti, S. Lloyd, L. Maccone, Advances in quantum metrology. Nat. Photonics 5, 222–229 (2011).

[14] M. Taylor, W. Bowen, Quantum metrology and its application in biology. Phys. Rep. 615, 1–59 (2016).

[15] V. Giovannetti, S. Lloyd, L. Maccone, J. Shapiro, Sub-Rayleigh-diffraction-bound quantum imaging. Phys. Rev. A 79, 013827 (2009).

[16] D.-Q. Xu, X.-B. Song, H.-G. Li, D.-J. Zhang, H.-B. Wang, J. Xiong, K. Wang, Experimental observation of sub-Rayleigh quantum imaging with a two-photon entangled source. Appl. Phys. Lett. 106, 171104 (2015).

[17] M. Unternährer, B. Bessire, L. Gasparini, M. Perenzoni, A. Stefanov, Super-resolution quantum imaging at the Heisenberg limit. Optica 5, 1150–1154 (2018).

[18] M. Mitchell, J. Lundeen, A. Steinberg, Super-resolving phase measurements with a multiphoton entangled state. Nature 429, 161–164 (2004). 15141206 10.1038/nature02493

[19] P. Walther, J.-W. Pan, M. Aspelmeyer, R. Ursin, S. Gasparoni, A. Zeilinger, De Broglie wavelength of a non-local four-photon state. Nature 429, 158–161 (2004). 15141205 10.1038/nature02552

[20] M. D’Angelo, M. Chekhova, Y. Shih, Two-photon diffraction and quantum lithography. Phys. Rev. Lett. 87, 013602 (2001). 11461466 10.1103/PhysRevLett.87.013602

[21] G. Xiang, B. Higgins, D. Berry, H. Wiseman, G. Pryde, Entanglement-enhanced measurement of a completely unknown optical phase. Nat. Photonics 5, 43–47 (2011).

[22] C. Degen, F. Reinhard, P. Cappellaro, Quantum sensing. Rev. Mod. Phys. 89, 035002 (2017).

[23] M. Napolitano, M. Koschorreck, B. Dubost, N. Behbood, R. Sewell, M. W. Mitchell, Interaction-based quantum metrology showing scaling beyond the Heisenberg limit. Nature 471, 486–489 (2011). 21430776 10.1038/nature09778

[24] P. Yin, X. Zhao, Y. Yang, Y. Guo, W.-H. Zhang, G.-C. Li, Y.-J. Han, B.-H. Liu, J.-S. Xu, G. Chiribella, G. Chen, C.-F. Li, G.-C. Guo, Experimental super-Heisenberg quantum metrology with indefinite gate order. Nat. Phys. 19, 1122–1127 (2023).

[25] J. Beltrán, A. Luis, Breaking the Heisenberg limit with inefficient detectors. Phys. Rev. A 72, 045801 (2005).

[26] S. Boixo, S. Flammia, C. Caves, J. Geremia, Generalized limits for single-parameter quantum estimation. Phys. Rev. Lett. 98, 090401 (2007). 17359140 10.1103/PhysRevLett.98.090401

[27] S. Roy, S. Braunstein, Exponentially enhanced quantum metrology. Phys. Rev. Lett. 100, 220501 (2008). 18643409 10.1103/PhysRevLett.100.220501

[28] Y. Yang, Memory effects in quantum metrology. Phys. Rev. Lett. 123, 110501 (2019). 31573225 10.1103/PhysRevLett.123.110501

[29] H. Defienne, B. Ndagano, A. Lyons, D. Faccio, Polarization entanglement-enabled quantum holography. Nat. Phys. 17, 591–597 (2021).

[30] Z. He, Y. Zhang, X. Tong, L. Li, L. Wang, Quantum microscopy of cells at the Heisenberg limit. Nat. Commun. 14, 2441 (2023). 37117176 10.1038/s41467-023-38191-4 PMC10147633

[31] T. Juffmann, B. B. Klopfer, T. L. Frankort, P. Haslinger, M. A. Kasevich, Multi-pass microscopy. Nat. Commun. 7, 12858 (2016). 27670525 10.1038/ncomms12858 PMC5052624

[32] C. Wagenknecht, C.-M. Li, A. Reingruber, X.-H. Bao, A. Goebel, Y.-A. Chen, Q. Zhang, K. Chen, J.-W. Pan, Experimental demonstration of a heralded entanglement source. Nat. Photonics 4, 549–552 (2010).

[33] H. Zhang, X.-M. Jin, J. Yang, H.-N. Dai, S.-J. Yang, T.-M. Zhao, J. Rui, Y. He, X. Jiang, F. Yang, G.-S. Pan, Z.-S. Yuan, Y. Deng, Z.-B. Chen, X.-H. Bao, S. Chen, B. Zhao, J.-W. Pan, Preparation and storage of frequency-uncorrelated entangled photons from cavity-enhanced spontaneous parametric downconversion. Nat. Photonics 5, 628–632 (2011).

[34] C. Couteau, Spontaneous parametric down-conversion. Contemp. Phys. 59, 291–304 (2018).

[35] P. R. Bevington, D. K. Robinson, J. M. Blair, A. J. Mallinckrodt, S. McKay, Data reduction and error analysis for the physical sciences. Comput. Phys. 7, 415–416 (1993).

[36] S. Daryanoosh, S. Slussarenko, D. Berry, H. Wiseman, G. Pryde, Experimental optical phase measurement approaching the exact Heisenberg limit. Nat. Commun. 9, 4606 (2018). 30389924 10.1038/s41467-018-06601-7 PMC6214903

[37] L.-Z. Liu, Y.-Z. Zhang, Z.-D. Li, R. Zhang, X.-F. Yin, Y.-Y. Fei, L. Li, N.-L. Liu, F. Xu, Y.-A. Chen, J.-W. Pan, Distributed quantum phase estimation with entangled photons. Nat. Photonics 15, 137–142 (2021).

[38] L. V. Wang, H.-i. Wu, *Biomedical Optics: Principles And Imaging* (John Wiley & Sons, 2007).

[39] L. Li, Z. Liu, X. Ren, S. Wang, V.-C. Su, M.-K. Chen, C. H. Chu, H. Y. Kuo, B. Liu, W. Zang, G. Chen, C.-F. Li, G.-C. Guo, Metalens-array–based high-dimensional and multiphoton quantum source. Science 368, 1487–1490 (2020). 32587020 10.1126/science.aba9779

[40] B. G. Oripov, D. S. Rampini, J. Allmaras, M. D. Shaw, S. W. Nam, B. Korzh, A. N. McCaughan, A superconducting nanowire single-photon camera with 400,000 pixels. Nature 622, 730–734 (2023). 37880435 10.1038/s41586-023-06550-2

[41] A. Valencia, G. Scarcelli, M. D’Angelo, Y. Shih, Two-photon imaging with thermal light. Phys. Rev. Lett. 94, 063601 (2005). 15783729 10.1103/PhysRevLett.94.063601

[42] J.-E. Oh, Y.-W. Cho, G. Scarcelli, Y.-H. Kim, Sub-Rayleigh imaging via speckle illumination. Opt. Lett. 38, 682–684 (2013). 23455264 10.1364/OL.38.000682 PMC4617630

[43] Z. He, Y. Zhang, X. Tong, L. Li, L. V. Wang, Heisenberg scaling quantum microscopy: Experiment and theory. arXiv:2303.04948 [quant-ph] (2025).

[44] Y. Shih, Quantum imaging. IEEE J. Sel. Top Quantum Electron. 13, 1016–1030 (2007).

[45] K. Dabov, A. Foi, V. Katkovnik, K. Egiazarian, Image denoising by sparse 3-D transform-domain collaborative filtering. IEEE Trans. Image Process. 16, 2080–2095 (2007). 17688213 10.1109/tip.2007.901238

[46] J. W. Goodman, *Introduction to Fourier Optics* (Roberts and Company Publishers, 2005).

[47] M. Born, E. Wolf, *Principles of Optics: Electromagnetic Theory of Propagation, Interference and Diffraction of Light* (Elsevier, 2013).

