# Supplementary material for: Above-twofold quantum super-resolution microscopy enabled by multiple idler passes with entangled biphotons
Source: Sci Adv. 2026 Jul 24;12(30):eaea9457. doi: 10.1126/sciadv.aea9457 (PMC13398480; doi:10.1126/sciadv.aea9457)
Supplement: Supplementary file 1 — Supplementary Notes S1 to S5 Figs. S1 to S17 Table S1 References [file sciadv.aea9457_sm.pdf]

Science Advances
AAAS

Supplementary Materials for

Above-twofold quantum super-resolution microscopy enabled by multiple idler passes with entangled biphotons

Xin Tong *et al.*

Corresponding author: Lihong V. Wang, lvw@caltech.edu

Sci. Adv. **12**, eaea9457 (2026)
DOI: 10.1126/sciadv.aea9457

This PDF file includes:

Supplementary Notes S1 to S5
Figs. S1 to S17
Table S1
References

Supplementary note 1: Coincidence estimation algorithm

The EMCCD captured a time-lapsed image stack for each object. At each frame (Fig. S2A), two half disks (i.e., the SPDC beam split by the prism) are shown. Prior to scanning the object, we estimate the center of the image $\mathbf{r}_c$ through a point-by-point cross-correlation scan from 50 frames without any object (details in Fig. S8 of Ref. (*30*)).

After locating $\mathbf{r}_c$, we place the object and acquire ∼2 million image frames. From the 3D (x-y-t) image stack, the intensities of two pixels in the left ($\mathbf{r}_1$, signal) and right ($\mathbf{r}_2$, idler) images are given by

$$I_1(i_t) = I_1^{\mathrm{coin}}(i_t) + I_1^{\mathrm{uncorr}}(i_t), \tag{S1}$$

$$I_2(i_t) = I_2^{\mathrm{coin}}(i_t) + I_2^{\mathrm{uncorr}}(i_t), \tag{S2}$$

where i_t denotes the frame index, I^{coin} the reading from the SPDC beam responsible for coincidence, and I^{uncorr} the reading from other sources (e.g., readout noise) that are uncorrelated with I^{coin}.

The temporal covariance between I_1 and I_2 is defined by

$$\mathrm{Cov}_t(I_1, I_2) = \frac{1}{N_t}\sum_{i_t}^{N_t}[I_1(i_t) - \overline{I_1}][I_2(i_t) - \overline{I_2}] = \overline{I_1 I_2} - \overline{I_1}\cdot\overline{I_2}, \tag{S3}$$

where N_t is the number of frames and the mean value is computed with respect to time. Eq. (S3) can be simplified to

$$\mathrm{Cov}_t(I_1, I_2) = \overline{I_1^{\mathrm{coin}} I_2^{\mathrm{coin}}} - \overline{I_1^{\mathrm{coin}}}\cdot\overline{I_2^{\mathrm{coin}}} + \overline{I_1^{\mathrm{uncorr}} I_2^{\mathrm{uncorr}}} - \overline{I_1^{\mathrm{uncorr}}}\cdot\overline{I_2^{\mathrm{uncorr}}}, \tag{S4}$$

while the first two terms represent the covariance of the SPDC signal, the last two terms represent the covariance of the detection noise:

$$\mathrm{Cov}_t(I_1, I_2) = \mathrm{Cov}_t(I_1^{\mathrm{coin}}, I_2^{\mathrm{coin}}) + \mathrm{Cov}_t(I_1^{\mathrm{uncorr}}, I_2^{\mathrm{uncorr}}). \tag{S5}$$

When $\mathbf{r}_1$ and $\mathbf{r}_2$ are selected so that $\mathbf{r}_1 + \mathbf{r}_2 = 2\mathbf{r}_c$, many more entangled photons appear in the temporal profiles I_1 and I_2 provided that the quantum efficiency of the detector is sufficiently high, leading to $I_1^{\mathrm{coin}} \approx I_2^{\mathrm{coin}}$. Conversely, when $\mathbf{r}_1 + \mathbf{r}_2' \neq 2\mathbf{r}_c$, I_1 and I_2' exhibit much less correlation (Fig. S2C), i.e., $\mathrm{Cov}_t(I_1^{\mathrm{coin}}, I_2^{\mathrm{coin}}) \gg \mathrm{Cov}_t(I_1^{\mathrm{coin}}, I_{2'}^{\mathrm{coin}})$.

Moreover, the covariance of the uncorrelated signal approximately vanishes. In Fig. S3 of Ref. (*30*), we have experimentally demonstrated that $\mathrm{Cov}_t(I_1^{\mathrm{coin}}, I_2^{\mathrm{coin}}) \gg \mathrm{Cov}_t(I_1^{\mathrm{uncorr}}, I_2^{\mathrm{uncorr}})$ when the average number of photons received per pixel is less than unity.

Further, as the coincidence intensity follows a Poisson distribution (*34*), for which the variance equals the mean, we have

$$\mathrm{Cov}_t(I_1, I_2) \approx \mathrm{Cov}_t(I_1^{\mathrm{coin}}, I_2^{\mathrm{coin}}) \approx \mathrm{Var}_t(I_1^{\mathrm{coin}}) = \overline{I_1^{\mathrm{coin}}}. \tag{S6}$$

We use the $\overline{I_1^{\mathrm{coin}}}$ to estimate the intensity correlation $G^{(2)}$. By repeating Eq. (S6) over all $\mathbf{r_1}$ in our field of view, we finally acquire the coincidence image.

Supplementary note 2: Quantification of effective numerical aperture (NA)

To experimentally measure our effective NA, we select three locations along the optical path, namely in the object plane (Fig. S10B), after the first imaging lens (f_1, Fig. S10C), and before the EMCCD (Fig. S10D). We use different strategies to compute the effective NA and reach similar results.

Between the two objectives, we measure the beam diameters at the focus (Pos 1) and at another axial (z) position (Pos 2) that is $z_{12} = 1.16$ mm from Pos 1. Due to the small focal point, the beam diameter in FWHM at Pos 1 is negligible. Therefore, the effective NA in the object plane can be expressed as

$$\mathrm{NA_{obj}} \approx \frac{\mathrm{FWHM}}{2z_{12}} = 0.18, \tag{S7}$$

leading to an Abbe diffraction limit (DL) of

$$\mathrm{DL_{obj}} = \frac{\lambda}{2\mathrm{NA_{obj}}} = 2.25\,\mu\mathrm{m}. \tag{S8}$$

Immediately after the first imaging lens (Pos 3), we measure the beam diameter and relay it before the EMCCD to form the exit pupil. The exit pupil has a diameter of $d_{\mathrm{XP}} = 2.30$ mm and is $z_{\mathrm{XP}} = 80$ mm from the EMCCD according to the imaging relation. Considering the magnification ratio M of the imaging system, we then compute the exit-pupil-related NA as

$$\mathrm{NA_{XP}} = \frac{M d_{\mathrm{XP}}}{2z_{\mathrm{XP}}} = 0.19, \tag{S9}$$

leading to a DL of

$$\mathrm{DL_{XP}} = \frac{\lambda}{2\mathrm{NA_{XP}}} = 2.13\,\mu\mathrm{m}. \tag{S10}$$

Before the EMCCD, we measure the beam diameters at two axial positions (Pos 4 and Pos 5) and measure their axial distance z_{45}. Therefore, the effective NA in the EMCCD plane can be expressed as

$$\mathrm{NA_{EMCCD}} = \frac{M(\mathrm{FWHM_4} - \mathrm{FWHM_5})}{2z_{45}} = 0.14, \tag{S11}$$

leading to a DL of

$$\mathrm{DL_{EMCCD}} = \frac{\lambda}{2\mathrm{NA_{EMCCD}}} = 2.89\,\mu\mathrm{m}. \tag{S12}$$

The NAs corresponding to the full width at 1/10 of maximum ($\mathrm{FW}\frac{1}{10}\mathrm{M}$) can be calculated similarly, which are much closer to the nominal NA = 0.4 which is given for planar-wave illumination on the full aperture. A summary of the NAs and the associated DLs are summarized in Supplementary table 1.

Supplementary note 3: Post-selection efficiency in SR2 and SR4

To implement quantum imaging, we first calibrate the experimental system with the EMCCD to locate the center of symmetry of the biphoton correlations. We then use this center to post-select photon counts corresponding to center-symmetric pairs of entangled photons, thereby reconstructing the entangled-photon contribution. This post-selection is essential for quantum imaging and for achieving resolution beyond the classical limit.

To support the theoretical derivation, we further analyzed the experimental data. This analysis yields two key observations that support the more restrictive post-selection inherent in the SR4 mechanism in comparison to SR2.

First, we observe a substantial reduction in coincidence counts for SR4 relative to SR2. As shown in Fig. S16, this reduction is greater than expected from the additional optical losses introduced by the triple-pass configuration alone. Quantitatively, this effect can be understood by comparing the effective post-selection area with the biphoton correlation area. For SR2, the post-selection efficiency is

$$\eta_{\mathrm{ps2}} = c\left(\frac{\Delta r_{\mathrm{SR2}}}{\Delta r_c}\right)^2, \tag{S13}$$

where c is a proportionality constant, Δr_{SR2} denotes the SR2 resolution arising from post-selection, and Δr_c denotes the biphoton correlation width. For SR4, the corresponding post-selection efficiency is

$$\eta_{\mathrm{ps4}} = c\left(\frac{\Delta r_{\mathrm{SR4}}}{\Delta r_c}\right)^2, \tag{S14}$$

where Δr_{SR4} denotes the SR4 resolution arising from post-selection. The ratio of the two post-selection efficiencies is predicted to be

$$\frac{\eta_{\mathrm{ps2}}}{\eta_{\mathrm{ps4}}} = \left(\frac{\Delta r_{\mathrm{SR2}}}{\Delta r_{\mathrm{SR4}}}\right)^2 = 4, \tag{S15}$$

which agrees closely with the experimental ratio of 3.7 ± 0.3 (Fig. S16D). We therefore interpret the excess loss as the first evidence of a more restrictive post-selection process in the SR4 measurement.

Second, we tested the robustness of the SR4 enhancement by varying the moving-average window size to introduce spatial blurring to the signal arm, the idler arm, or both arms in the SR4 dataset (Figs. S17A and B).

In Fig. S17C, we compare the classical imaging resolutions before and after convolution; their ratio varies monotonically. Modeling the moving-average operation as a convolution with a top-hat kernel of width w, we approximate the resulting blur by variance addition under convolution. Because a top-hat kernel has variance $\sigma^2 = w^2/12$, conversion to an equivalent Gaussian FWHM using FWHM = $2\sqrt{2\ln 2}\,\sigma$ gives $\mathrm{FWHM}_{\mathrm{conv}}(w) \approx \sqrt{r_0^2 + \frac{2\ln 2}{3}\,w^2} \approx \sqrt{r_0^2 + 0.46\,w^2}$, where $r_0 \sim 4$ pixels denotes the pre-convolution classical resolution.

In Fig. S17D, convolving the signal arm causes the enhancement factor to drop rapidly from 4 to 2 after the first convolution, remain near 2 over a substantial range of kernel sizes, and then rise again. The initial drop reflects the higher spatial resolving power of SR4 relative to SR2. The eventual rise reflects ghost imaging: once classical imaging no longer provides sufficient resolution, the post-convolution quantum imaging can still recover spatial information.

In Figs. S17E and F, enlarging the idler-arm convolution kernel without and with blurring the signal-arm image causes the quantum resolution to degrade steadily toward the classical limit. This trend is expected because coincidence detection through the idler arm becomes progressively less informative as the idler data is blurred. Comparison of Figs. S17C and E further suggests that the signal photon carries more imaging information than the idler photon and is therefore more sensitive to blurring. Taken together, Fig. S17 supports the conclusion that the observed fourfold resolution enhancement in SR4 is not an artifact due to noise.

These two observations, the intrinsic coincidence loss and the sensitivity to spatial blurring, provide strong experimental support for our theoretical model. They confirm that the larger enhancement observed in SR4 arises from the more restrictive post-selection required to preserve transverse correlations across multiple passes.

Supplementary note 4: Theory for super-resolution quantum coincidence imaging

We compare classical and quantum imaging of a pinhole object through a thin lens, for simplicity, with a magnification of unity. The image of the pinhole yields the point spread function (PSF), defining the spatial resolution. The key difference arises from phase accumulation and quantum interference in the image plane. Because of the approximate transverse shift invariance (*46*), the pinhole is conveniently placed on the optical axis without losing generality.

Classical imaging

As shown in Fig. S15A, a single photon passes through a thin lens. The classical PSF is the Airy disk determined by the Fourier transform of the circular exit pupil (*46, 47*):

$$I_{\mathrm{CI}}(r) = \left| \int_{|\mathbf{q}| \le q_{\max}} e^{i\mathbf{q}\cdot\mathbf{r}} d^2\mathbf{q} \right|^2 \propto \left[\mathrm{somb}\left(q_{\max} r\right)\right]^2, \tag{S16}$$

where $\mathbf{r}$ denotes the transverse position relative to the optical axis and $\mathbf{q}$ denotes the transverse wave vector within the following limit (see Eq. S22):

$$q_{\max} = k\,\mathrm{NA}, \quad k = 2\pi/\lambda,\ \mathrm{NA} \le 1. \tag{S17}$$

NA denotes the numerical aperture of the circular exit pupil. The classical resolution limit is approximately

$$\Delta r_{\mathrm{CI}} = \frac{\lambda}{2\,\mathrm{NA}}. \tag{S18}$$

Quantum imaging

In SR2, as shown in Fig. S15B, two entangled photons pass through identical lenses and are detected jointly. In SR4, the idler photon undergoes three passes through the optics, while the signal photon does only once, as shown in Fig. S15C. The following derivation was inspired by Shih (*44*).

We begin with a general entangled state of two photons in the object and virtual-object planes of the two arms:

$$|\Psi_{\mathrm{in}}\rangle = \int_{|\mathbf{q}| \le q_{\mathrm{src}}} d^2\mathbf{q}\, \Phi(\mathbf{q})\, \hat{a}_1^\dagger(\mathbf{q})\, \hat{a}_2^\dagger(-\mathbf{q})\ |0\rangle \tag{S19}$$

The transverse-momentum amplitude $\Phi(\mathbf{q})$ is supported on $|\mathbf{q}| \le q_{\mathrm{src}}$, $\hat{a}_i^\dagger(\mathbf{q})$ creates a photon of transverse momentum $\mathbf{q}$ in arm $i = 1, 2$ while satisfying

$$\left[\hat{a}_i(\mathbf{q}_i), \hat{a}_j^\dagger(\mathbf{q}_j)\right] = \delta_{ij}\, \delta^{(2)}(\mathbf{q}_i - \mathbf{q}_j). \tag{S20}$$

Because the imaging optics in each arm admit only momenta within q_{pupil}, we define a projector

$$\hat{P} = \int_{|\mathbf{q}| \le q_{\mathrm{pupil}}} d^2\mathbf{q}\, \big|\mathbf{q}, -\mathbf{q}\big\rangle\big\langle\mathbf{q}, -\mathbf{q}\big| \quad , \quad \big|\mathbf{q}_1, \mathbf{q}_2\big\rangle = \hat{a}_1^\dagger(\mathbf{q}_1)\, \hat{a}_2^\dagger(\mathbf{q}_2)\ |0\rangle\,. \tag{S21}$$

Acting on $|\Psi_{\mathrm{in}}\rangle$, $\hat{P}$ truncates support within the following limit (see Eq. S17):

$$q_{\max} = \min(q_{\mathrm{src}}, q_{\mathrm{pupil}}). \tag{S22}$$

Consequently, we obtain

$$|\Psi_{\mathrm{img}}\rangle = \hat{P}\ |\Psi_{\mathrm{in}}\rangle = \int_{|\mathbf{q}| \le q_{\max}} d^2\mathbf{q}\, \Phi(\mathbf{q})\, \hat{a}_1^\dagger(\mathbf{q})\, \hat{a}_2^\dagger(-\mathbf{q})\ |0\rangle\,. \tag{S23}$$

In the image plane, the positive-frequency field at transverse position $\mathbf{r}$ is assumed to accumulate transverse phase through coincidence detection:

$$\hat{E}_i^{(+)}(\mathbf{r}) = \int d^2\mathbf{q}\, \hat{a}_i(\mathbf{q})\, e^{i n_i (\mathbf{q}\cdot\mathbf{r})}. \tag{S24}$$

The number of passes in each arm is denoted by n_i. Specifically, $n_1 = 1$, and $n_2 = n = 1, 3$ for SR2 and SR4, respectively.

This equation, when applied to the idler arm, is an ansatz to describe the post-selected idler field operator after coincidence measurement. The central idea is that post-selection favors photon pairs for which the idler photon remains transversely correlated with the signal photon throughout the additional passes, thereby allowing the corresponding idler-side transverse phases to combine constructively in the detected two-photon probability amplitude (see the next subsection for more details). In other words, post-selection reconstructs the entangled state. Accordingly, this equation should be understood not as describing classical idler-beam propagation, but as an effective positive-frequency field operator for two-photon coincidence detection.

This ansatz is supported by the experimentally validated scaling of post-selection efficiency, which is consistent with a more restrictive spatial post-selection governed by the ratio between the resolution size and the biphoton correlation width (Supplementary Note 3). We emphasize that the experimental observations reported here are independent of the ansatz.

The joint two-photon detection operator is $\hat{\Psi}(\mathbf{r}_1, \mathbf{r}_2) = \hat{E}_1^{(+)}(\mathbf{r}_1)\,\hat{E}_2^{(+)}(\mathbf{r}_2)$. The two-photon wavefunction gives the probability amplitude to detect one photon at $\mathbf{r}_1$ in arm 1 and one at $\mathbf{r}_2$ in arm 2:

$$\psi(\mathbf{r}_1, \mathbf{r}_2) = \langle 0|\ \hat{\Psi}(\mathbf{r}_1, \mathbf{r}_2)\ |\Psi_{\mathrm{img}}\rangle = \langle 0|\ \hat{E}_1^{(+)}(\mathbf{r}_1)\,\hat{E}_2^{(+)}(\mathbf{r}_2) \int_{|\mathbf{q}|\le q_{\max}} d^2\mathbf{q}\,\Phi(\mathbf{q})\,\hat{a}_1^\dagger(\mathbf{q})\,\hat{a}_2^\dagger(-\mathbf{q})\ |0\rangle\,. \tag{S25}$$

Substituting the field operators yields

$$\begin{aligned}\psi(\mathbf{r}_1, \mathbf{r}_2) = \int d^2\mathbf{q}_1\, d^2\mathbf{q}_2\, e^{i(\mathbf{q}_1\cdot\mathbf{r}_1 + n\mathbf{q}_2\cdot\mathbf{r}_2)} &\int_{|\mathbf{q}|\le q_{\max}} d^2\mathbf{q}\,\Phi(\mathbf{q}) \\ &\times \langle 0|\ \hat{a}_1(\mathbf{q}_1)\,\hat{a}_2(\mathbf{q}_2)\,\hat{a}_1^\dagger(\mathbf{q})\,\hat{a}_2^\dagger(-\mathbf{q})\ |0\rangle\,.\end{aligned} \tag{S26}$$

Using

$$\hat{a}_1(\mathbf{q}_1)\,\hat{a}_1^\dagger(\mathbf{q})\,|0\rangle = \delta^{(2)}(\mathbf{q}_1 - \mathbf{q})\,|0\rangle \tag{S27}$$

and

$$\hat{a}_2(\mathbf{q}_2)\,\hat{a}_2^\dagger(-\mathbf{q})\,|0\rangle = \delta^{(2)}(\mathbf{q}_2 + \mathbf{q})\,|0\rangle\,, \tag{S28}$$

we reach

$$\psi(\mathbf{r}_1, \mathbf{r}_2) = \int_{|\mathbf{q}|\le q_{\max}} d^2\mathbf{q}\,\Phi(\mathbf{q})\,e^{i(\mathbf{q}\cdot\mathbf{r}_1 - n\mathbf{q}\cdot\mathbf{r}_2)} = \int_{|\mathbf{q}|\le q_{\max}} d^2\mathbf{q}\,\Phi(\mathbf{q})\,e^{i\,\mathbf{q}\cdot(\mathbf{r}_1 - n\mathbf{r}_2)}. \tag{S29}$$

If $\Phi(\mathbf{q}) \equiv 1$ over the disk, we obtain

$$\psi(\mathbf{r}_1, \mathbf{r}_2) = \int_{|\mathbf{q}|\le q_{\max}} d^2\mathbf{q}\,e^{i\mathbf{q}\cdot(\mathbf{r}_1 - n\mathbf{r}_2)}. \tag{S30}$$

For center-symmetric coincidence detection, we set $\mathbf{r}_1 = \mathbf{r}$, $\mathbf{r}_2 = -\mathbf{r}$. Then

$$\psi(\mathbf{r}, -\mathbf{r}) = \int_{|\mathbf{q}|\le q_{\max}} d^2\mathbf{q}\,e^{i(1+n)\mathbf{q}\cdot\mathbf{r}}. \tag{S31}$$

The coincidence detection is described by the second-order correlation function:

$$G^{(2)}_{\mathrm{SR}}(\mathbf{r}, -\mathbf{r}) = |\psi(\mathbf{r}, -\mathbf{r})|^2 \propto \left[\mathrm{somb}\left((1+n)\,q_{\max} r\right)\right]^2. \tag{S32}$$

Hence, the quantum PSF is an Airy pattern that is narrowed by a factor of $1 + n$.

$$\Delta r_{\mathrm{SR}} = \frac{1}{1+n}\Delta r_{\mathrm{CI}} = \frac{\lambda}{2(1+n)\,\mathrm{NA}}. \tag{S33}$$

This confirms that the coincidence imaging achieves super-resolution consistent with the experimental observations, arising from transverse phase accumulation in the two-photon wavefunction.

Rationale for Eq. S24

In this subsection, we provide a more detailed phenomenological justification for the ansatz introduced in Eq. (S24). The argument below should be understood as an effective post-selection model rather than a first-principles derivation.

The biphoton state generated by SPDC, written in coordinates of the object plane, which is taken to be the momentum plane of the source, is

$$|\Psi\rangle = \int d^2\boldsymbol{r}_{\mathrm{st}}^{(s)}\, \Phi(\boldsymbol{r}_{\mathrm{st}}^{(s)})\, \hat{a}_s^{\dagger}(\boldsymbol{r}_{\mathrm{st}}^{(s)})\, \hat{a}_i^{\dagger}(-\boldsymbol{r}_{\mathrm{st}}^{(s)})\, |\mathrm{vac}\rangle\,, \tag{S34}$$

where $\boldsymbol{r}_{\mathrm{st}}^{(s)}$ denotes the transverse coordinates at the starting (object) plane in the signal arm. We will assume Φ varies slowly over the object support in later derivation for brevity.

The signal-arm electric field operator is written as

$$\hat{E}_s^{(+)}(\boldsymbol{r}_{\mathrm{ar}}^{(s)}) \propto \int d^2\boldsymbol{r}_l^{(s)} d^2\boldsymbol{r}_{\mathrm{st}}^{(s)}\, h(\boldsymbol{r}_{\mathrm{st}}^{(s)}, \boldsymbol{r}_l^{(s)}, \boldsymbol{r}_{\mathrm{ar}}^{(s)}) t(\boldsymbol{r}_{\mathrm{st}}^{(s)})\, \hat{a}_s(\boldsymbol{r}_{\mathrm{st}}^{(s)}). \tag{S35}$$

Here, $\boldsymbol{r}_l^{(s)}$ denotes the transverse coordinates at the lens plane in the signal arm, and t denotes the object transmission coefficient. The integral over $\boldsymbol{r}_l^{(s)}$ is taken over the aperture of the lens. The function h describes propagation through free space and the imaging lens in an equivalent single-lens imaging system, given by (*46*)

$$h\left(\boldsymbol{r}_{\mathrm{st}}, \boldsymbol{r}_l, \boldsymbol{r}_{\mathrm{ar}}\right) \propto e^{-i\frac{k}{2f}(\boldsymbol{r}_{\mathrm{st}}+\boldsymbol{r}_{\mathrm{ar}})\cdot\boldsymbol{r}_l}, \tag{S36}$$

where f denotes the focal length of the imaging lens, and unit magnification is assumed for simplicity.

The three-pass idler electric field operator before post-selection is

$$\hat{E}_i^{(+)}(\boldsymbol{r}_{\mathrm{ar}}^{(i,3)}) \propto \int \left[\prod_{m=3}^{1} d^2\boldsymbol{r}_l^{(i,m)} d^2\boldsymbol{r}_{\mathrm{st}}^{(i,m)}\right] \left[\prod_{m=3}^{1} h\left(\boldsymbol{r}_{\mathrm{st}}^{(i,m)}, \boldsymbol{r}_l^{(i,m)}, \boldsymbol{r}_{\mathrm{ar}}^{(i,m)}\right)\right]\, \hat{a}_i(\boldsymbol{r}_{\mathrm{st}}^{(i,1)}). \tag{S37}$$

Here, $\boldsymbol{r}_{\mathrm{st}}^{(i,m)}$, $\boldsymbol{r}_l^{(i,m)}$, and $\boldsymbol{r}_{\mathrm{ar}}^{(i,m)}$ denote the transverse coordinates on the starting plane, lens plane, and arriving plane, respectively, during the m-th pass. In the first pass, the starting plane coincides with the virtual object plane, whereas in the third pass, the arriving plane coincides with the detection plane. The integral over $\boldsymbol{r}_l^{(i,m)}$ is taken over the aperture of the lens. Because of reflection, the starting coordinates of the $(m+1)$-th pass equal the arriving coordinates of the m-th pass, i.e., $\boldsymbol{r}_{\mathrm{st}}^{(i,2)} = \boldsymbol{r}_{\mathrm{ar}}^{(i,1)}$ and $\boldsymbol{r}_{\mathrm{st}}^{(i,3)} = \boldsymbol{r}_{\mathrm{ar}}^{(i,2)}$. Note that, in the absence of any constraints on the integration variables, the integral reduces to an ordinary single-pass imaging propagator.

The coincidence signal is given by the second-order correlation function:

$$\begin{aligned} G^{(2)}(\boldsymbol{r}_{\mathrm{ar}}^{(s)}, \boldsymbol{r}_{\mathrm{ar}}^{(i,3)}) &= \langle\Psi|\hat{E}_i^{(-)}(\boldsymbol{r}_{\mathrm{ar}}^{(i,3)})\hat{E}_s^{(-)}(\boldsymbol{r}_{\mathrm{ar}}^{(s)})\hat{E}_s^{(+)}(\boldsymbol{r}_{\mathrm{ar}}^{(s)})\hat{E}_i^{(+)}(\boldsymbol{r}_{\mathrm{ar}}^{(i,3)})|\Psi\rangle \\ &= \left|\langle\mathrm{vac}|\hat{E}_s^{(+)}(\boldsymbol{r}_{\mathrm{ar}}^{(s)})\hat{E}_i^{(+)}(\boldsymbol{r}_{\mathrm{ar}}^{(i,3)})|\Psi\rangle\right|^2. \end{aligned} \tag{S38}$$

In SR2, the reconstructed entangled state gives rise to transverse correlations between the signal and idler arms at both the object and lens planes (*44*), given by

$$\delta^{(2)}\big(\boldsymbol{r}_{\mathrm{st}}^{(i,1)} + \boldsymbol{r}_{\mathrm{st}}^{(s)}\big),\ \delta^{(2)}\big(\boldsymbol{r}_l^{(i,1)} + \boldsymbol{r}_l^{(s)}\big). \tag{S39}$$

The first delta function represents an anti-correlation because the object plane corresponds to the momentum plane of the source, where the SPDC photon pairs exhibit transverse momentum anti-correlation. The second delta function represents an anti-correlation in the lens plane arising from the center-symmetric post-selection imposed in the detection planes.

In SR4, the idler photon propagates through the same imaging system three times. The first pass is identical to that in SR2, so the signal–idler correlations in Eq. (S39) remain applicable. After the first pass, the idler field is reflected and routed through the imaging system twice more. These additional passes remain unitary; however, diffraction accumulated during the longer propagation can reduce the

effective transverse overlap between the signal and idler photons at the single-photon level, consistent with the experimentally observed fourfold reduction in the SR4 post-selection efficiency (Fig. S16).

We therefore adopt the following hypothesis: the coincidence measurement, performed at center-symmetric positions about the correlation point, post-selects a subset of SR2 photon pairs for which the signal–idler transverse correlations are preserved throughout the entire three-pass propagation. This hypothesis can be mathematically expressed using the following delta-function constraints:

$$\delta^{(2)}\big(\boldsymbol{r}_{\text{ar}}^{(i,1)} + \boldsymbol{r}_{\text{ar}}^{(s)}\big),\ \delta^{(2)}\big(\boldsymbol{r}_{l}^{(i,2)} + \boldsymbol{r}_{l}^{(s)}\big),\ \delta^{(2)}\big(\boldsymbol{r}_{\text{st}}^{(i,3)} + \boldsymbol{r}_{\text{st}}^{(s)}\big),\ \delta^{(2)}\big(\boldsymbol{r}_{l}^{(i,3)} + \boldsymbol{r}_{l}^{(s)}\big). \tag{S40}$$

Inserting Eqs. (S34), (S35), (S37), (S39), and (S40) into Eq. (S38) and evaluating at center-symmetric positions yield

$$G^{(2)}(\boldsymbol{r}_{\text{ar}}^{(s)}, \boldsymbol{r}_{\text{ar}}^{(i,3)} = -\boldsymbol{r}_{\text{ar}}^{(s)}) \propto \left| \int d^2\boldsymbol{r}_{\text{st}}^{(s)} \int_{\mathcal{R}} d^2\boldsymbol{r}_{l}^{(s)}\, t(\boldsymbol{r}_{\text{st}}^{(s)})\, h(\boldsymbol{r}_{\text{st}}^{(s)}, \boldsymbol{r}_{l}^{(s)}, \boldsymbol{r}_{\text{ar}}^{(s)}) \Big[h(-\boldsymbol{r}_{\text{st}}^{(s)}, -\boldsymbol{r}_{l}^{(s)}, -\boldsymbol{r}_{\text{ar}}^{(s)})\Big]^3 \right|^2. \tag{S41}$$

The appearance of the $[h]^3$ term indicates that, within the post-selected subset, the phases associated with the three idler propagators add constructively.

Further, from Eq. (S36), we obtain

$$G^{(2)}(\boldsymbol{r}_{\text{ar}}^{(s)}, \boldsymbol{r}_{\text{ar}}^{(i,3)} = -\boldsymbol{r}_{\text{ar}}^{(s)}) \propto \left| \int d^2\boldsymbol{r}_{\text{st}}^{(s)}\, t(\boldsymbol{r}_{\text{st}}^{(s)})\, \text{somb}\Big[4q_{\max}\left|\boldsymbol{r}_{\text{st}}^{(s)} + \boldsymbol{r}_{\text{ar}}^{(s)}\right|\Big] \right|^2. \tag{S42}$$

The somb function arises from integrating over the circular lens aperture $\mathcal{R}$ of radius R. In this single-lens unit-magnification setup, $q_{\max} = \frac{Rk}{2f}$. Setting $t(\boldsymbol{r}_{\text{st}}^{(s)}) = \delta^{(2)}(\boldsymbol{r}_{\text{st}}^{(s)})$ reduces Eq. (S42) to Eq. (S32) in the previous subsection. Within this idealized model, the factor of 4 in the argument corresponds to a fourfold narrowing of the point-spread function relative to classical imaging.

In summary, in SR4, center-symmetric coincidence is hypothesized to post-select the subset of idler photons that are correlated with the signal photon throughout the propagation. Under this assumption, the phase accumulated in the successive idler propagators can combine constructively. The ansatz in Eq. (S24), introduced in the previous subsection, provides a simplified representation of this constructive phase accumulation, resulting in fourfold resolution enhancement.

Supplementary note 5: Far-field anti-correlation measurement

We studied the effect of multiple passes on the entanglement using a separate experiment. We first used a single pass in the idler arm to find the center of correlation between the images from the two arms. Then, we switched to three passes in the idler arm and used the same center to construct the correlation image. The experiment demonstrated anti-correlation peaks in single and triple passes (Fig. S13). This observation shows that the transverse position correlation on the detection plane is maintained even after traversing the idler photon multiple times.

Supplementary figures

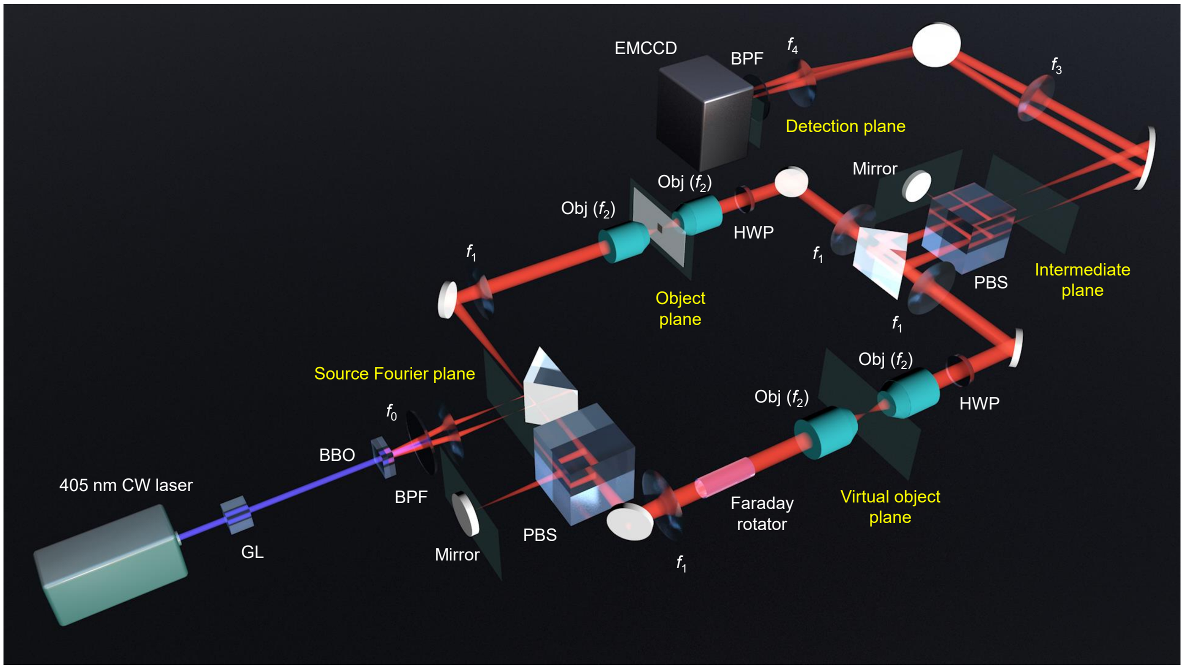

Fig. S1: **Detailed schematic of the experimental setup.** CW: continuous wave; GL: Glan–Laser polarizer; BBO: β-barium borate crystals; BPF: band-pass filter; PBS: polarizing beam splitter; Obj: objective; HWP: half-wave plate; EMCCD: electron multiplying charge-coupled device camera.

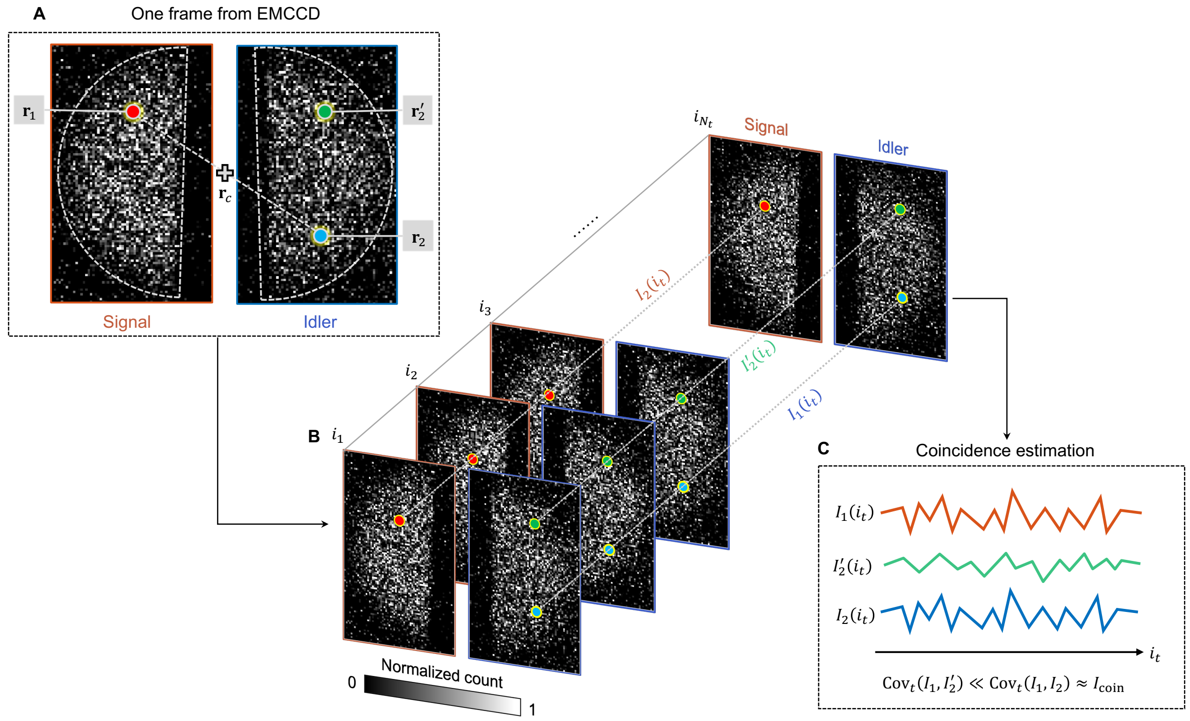

Fig. S2: **Schematic of the coincidence estimation algorithm.** (**A**) Single-frame EMCCD image of the SPDC beam with no object. The beam symmetry center ($\mathbf{r}_c$, cross) is calibrated based on the point-by-point scan of the covariance between the two half disks. Given the point $\mathbf{r_1}$ in the signal arm, $\mathbf{r_2}$ is the symmetric point in the idler arm while $\mathbf{r_2}'$ is another uncorrelated point. (**B**) Time-lapsed EMCCD image frames. The EMCCD readings on each pixel form a time sequence $I(i_t)$ for coincidence estimation. (**C**) Coincidence estimation. Temporal covariances are computed between the symmetric points as estimations of coincidence counts.

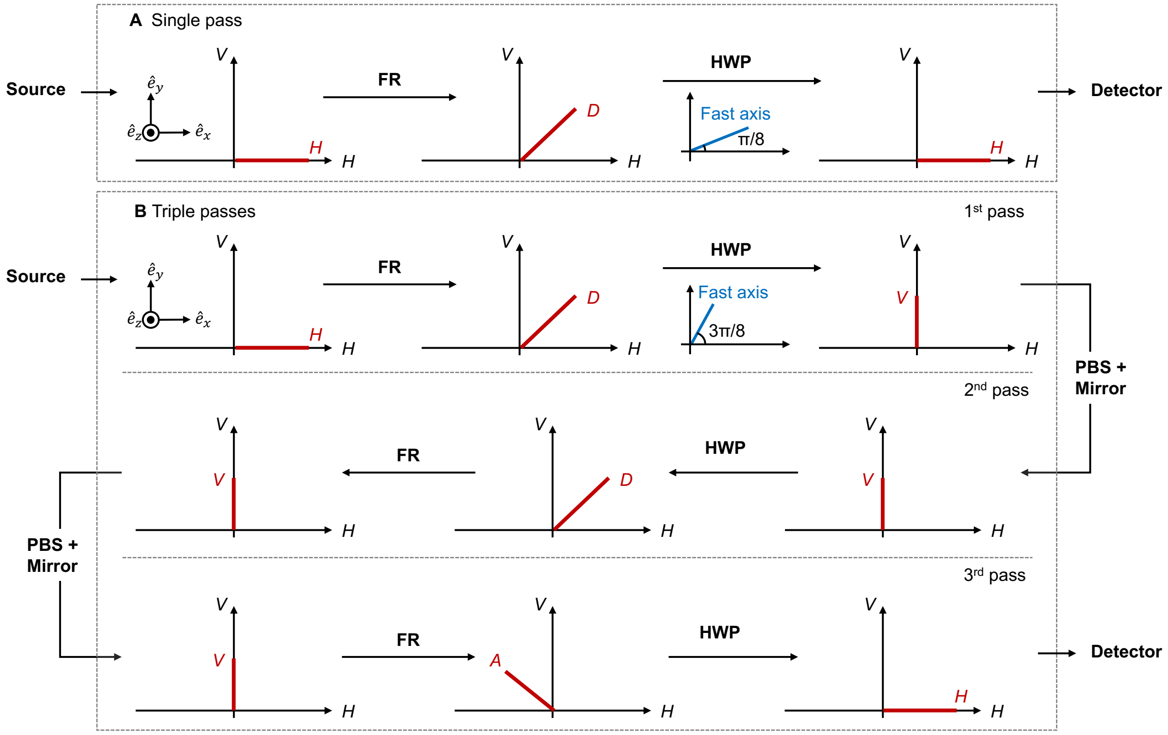

Fig. S3: **Polarization-state transformations in the idler arm.** (**A**) Single-pass and (**B**) triple-pass configurations. FR: Faraday rotator; HWP: half-wave plate; PBS: polarizing beam splitter. The coordinates are defined in Fig. 1.

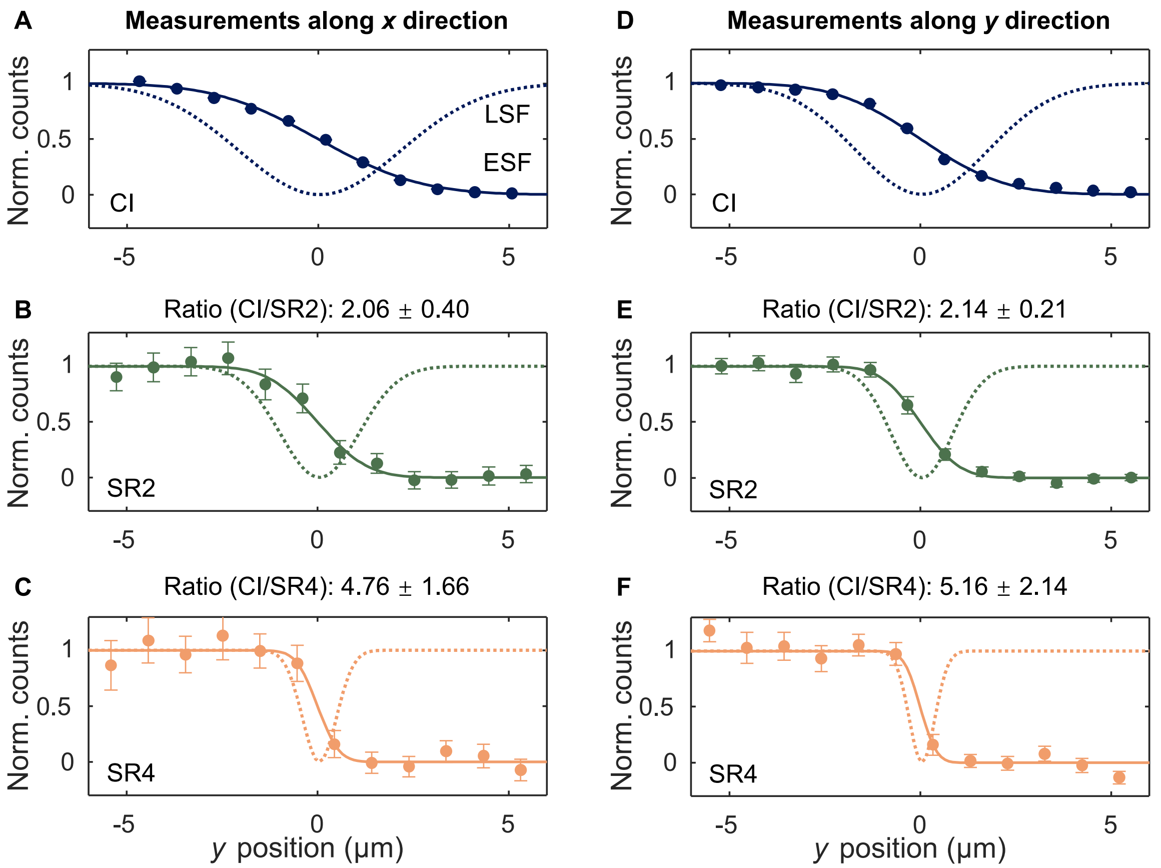

Fig. S4: **Quantification of the spatial resolutions along the x and y axes.** (**A**–**C**) Edge spread functions (ESFs) and line spread functions (LSFs) along x of the (**A**) CI, (**B**) SR2, and (**C**) SR4 images measured at the z positions marked by black circles in Fig. 2**A**. (**D**–**F**) ESFs and LSFs along y of the (**D**) CI, (**E**) SR2, and (**F**) SR4 images measured at the z positions marked by black box in Fig. 2**E**. The ESFs are from fitting the experimental data, each plotted as the mean ± standard error of the mean ($n = 30$). Norm, normalized.

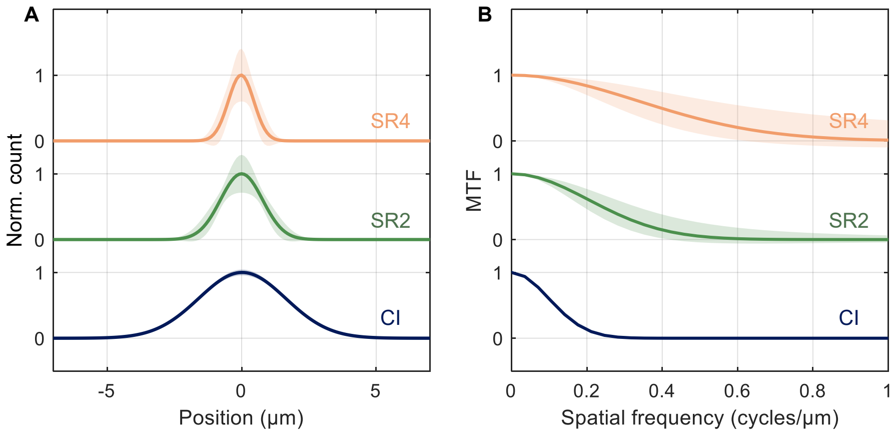

Fig. S5: **Quantification of the spatial resolutions using PSFs and MTFs.**(**A**) Normalized line spread functions (LSFs) of the CI, SR2, and SR4 obtained by fitting the measured edge spread functions (ESFs) to an error-function model. Shaded bands represent standard deviations. (**B**) Corresponding modulation transfer functions (MTFs) derived from the fitted LSFs using Fourier analysis. Shaded regions indicate standard deviations.

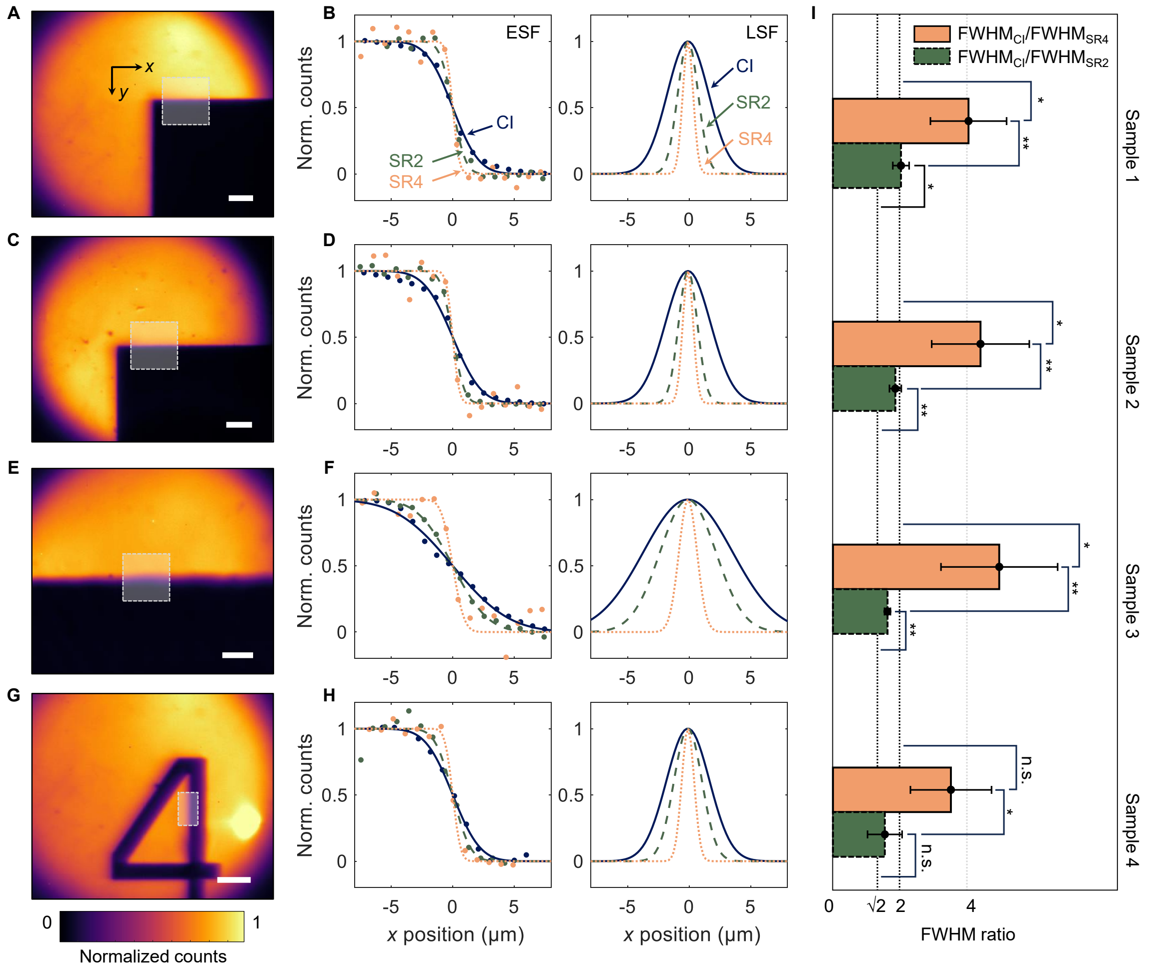

Fig. S6: **Repeated measurements with different samples.** (**A**) CI image of a corner of USAF 1951 resolution target as shown in Fig. 2**A**. (**B**) Raw and fitted edge spread functions (ESFs, left) and line spread functions (LSFs, right) of CI, SR2, and SR4 of the edge marked by dashed box in a. (**C**) CI image and (**D**) the associated ESFs and LSFs of another corner of USAF 1951 resolution target. (**E**) CI image and (**F**) the associated ESFs and LSFs of a knife edge. (**G**) CI image and (**H**) the associated ESFs and LSFs of the number "4" of the USAF target. (**I**) Resolution enhancement observed across four different samples. The p-values are obtained from one-sided t-tests. For samples 1 and 2, $n = 30$. For sample 3, $n = 50$. For sample 4, $n = 15$. $^{*}p < 0.05$, $^{**}p < 0.01$, n.s., $p > 0.05$. Scale bars, 10 μm.

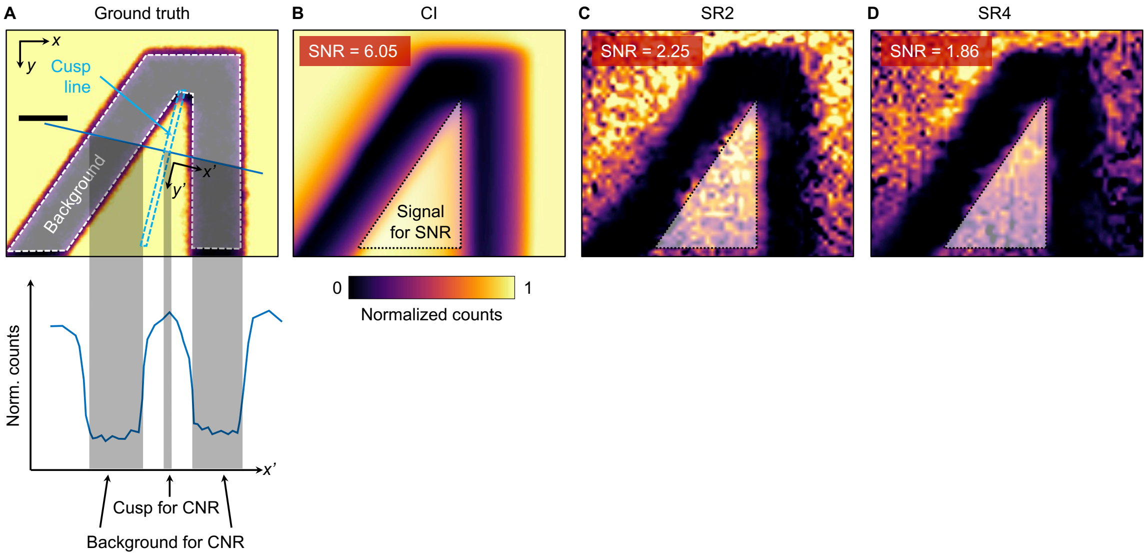

Fig. S7: **Contrast-to-noise ratio (CNR) versus signal-to-noise ratio (SNR) quantification.** (**A**) Ground-truth (GT) image of the number "4" in group 3 of a USAF 1951 resolution target. The CNR at a chosen y' position is calculated along the x' axis (blue solid line), where the signal and background are marked by the black arrows below. The region used to quantify the signal is marked as the cusp line. (**B**) CI, (**C**) SR2, and (**D**) SR4 images of the same field of view and the quantified SNR. The regions selected as "Background" (for CNR), "cusp" (for CNR), and "Signal" (for SNR) are marked by white dashed line, blue dashed line, and black dotted lines, respectively.

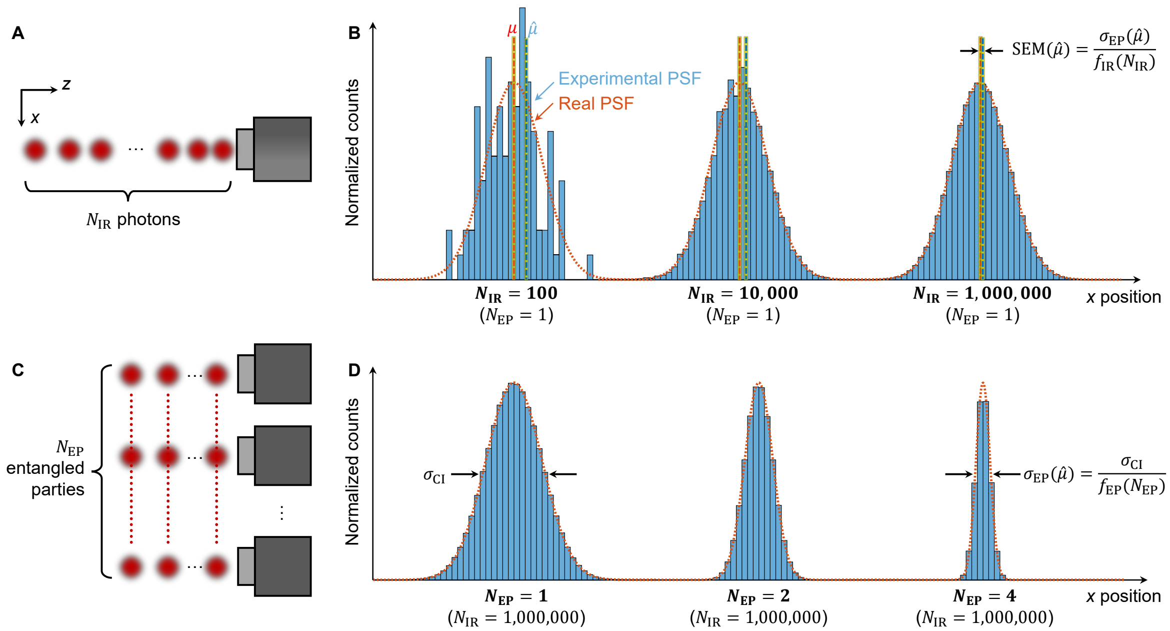

Fig. S8: **Simulated point spread function (PSF) and photon counting statistics.** (**A**) Schematic of sending N_{IR} photons to the detector. (**B**) Experimental PSF (in the form of photon counting histogram) versus real PSF with increasing N_{IR}. The standard error of the mean (SEM) describes the estimation of localizing the Gaussian mean μ and becomes lower as a function of N_{IR}. (**C**) Schematic of sending N_{EP} entangled photons to detectors. (**D**) Experimental PSF (in the form of photon counting histogram) versus real PSF with increasing N_{EP}. The Gaussian peak becomes narrower (i.e., the standard deviation σ gets smaller) as a function of N_{EP}. CI, Classical imaging.

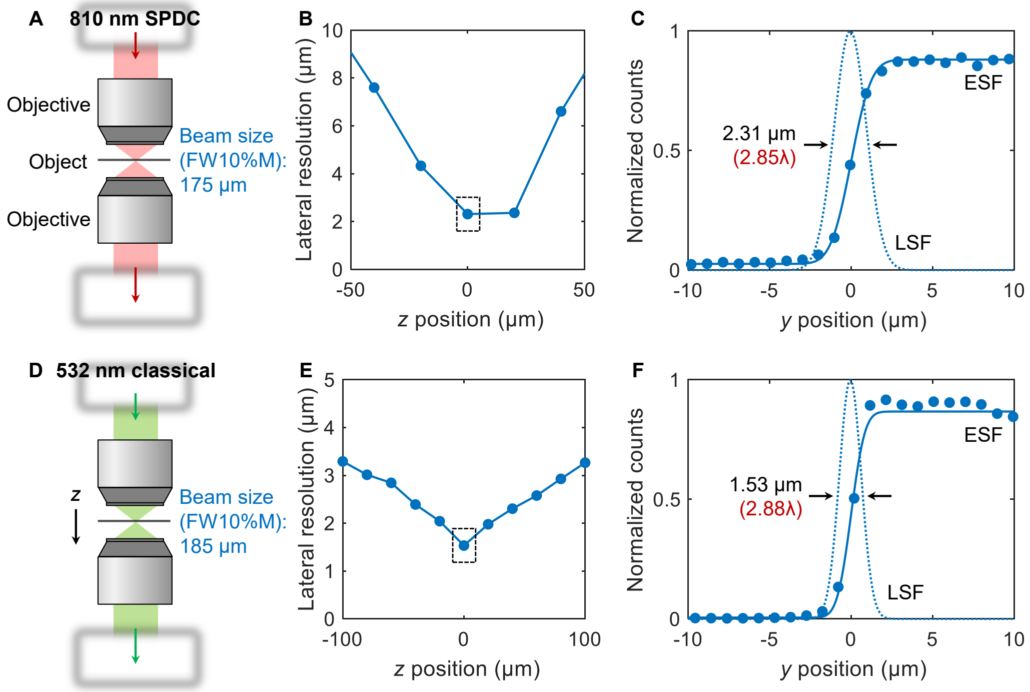

Fig. S9: **Resolution quantifications of CI images using SPDC beam and coherent laser illumination under the same NA.** (**A**) Schematic of the CI setup using an 810 nm SPDC beam. (**B**) Experimental lateral resolution versus z. (**C**) Raw (dots) and fitted (solid line) edge spread functions (ESFs) and line spread function (LSF, dotted line) along y measured at the z position marked by the black dashed box in (**B**). (**D**) Schematic of the CI setup using a 532 nm classical CW laser beam. (**E**) Experimental lateral resolution versus z. (**F**) Raw (dots) and fitted (solid line) edge spread functions (ESFs) and line spread function (LSF, dotted line) along y measured at the z position marked by the black dashed box in (**E**). FW10%M, full-width-at-10% maximum.

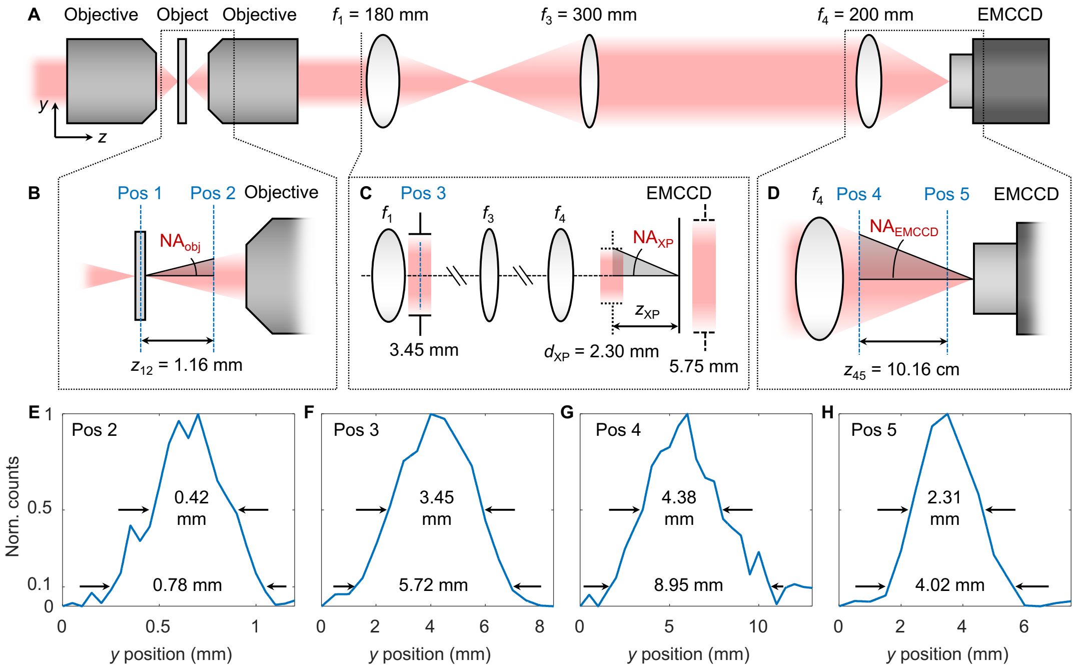

Fig. S10: **Beam size and numerical aperture analysis of the CI setup.** (**A**) Simplified schematic of the CI setup from the objectives to the EMCCD. (**B**) Close-up view between the two objectives illustrating the measurement of the objective-side NA. (**C**) Simplified schematic from lens f_1 to the EMCCD illustrating the measurement of the detection NA from the exit pupil (XP). (**D**) Close-up view between the last lens (f_4) and the EMCCD illustrating the measurement of the detection-side NA. (**E**–**H**) Beam profile measurement along the blue dashed lines in (**B**–**D**). Full widths at half maxima and 10% maxima are marked in the middle and at the bottom respectively.

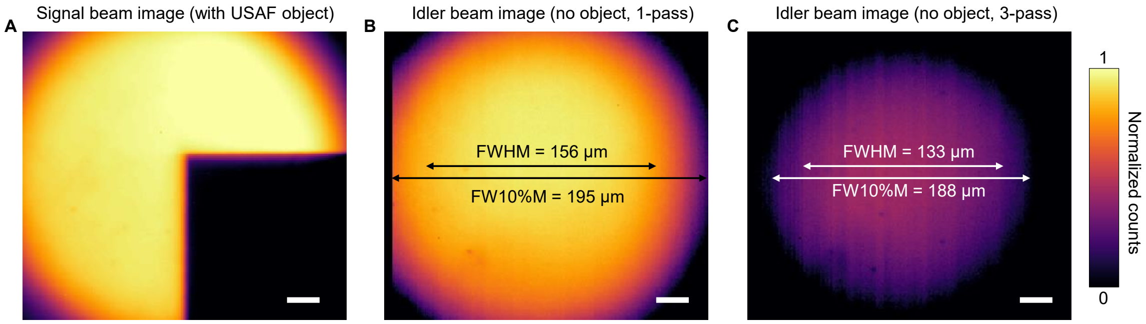

Fig. S11: **CI images from the signal and idler arms.** (**A**) CI image from the signal arm with the USAF 1951 resolution target in the object plane for ESF estimation. (**B**–**C**) CI images from the single-pass (**B**) and triple-pass (**C**) idler arm with no object. Note that in our imaging experiment, only the signal arm contains the object; the idler arm is always object-free. All images are normalized according to the maximum and minimum of (**A**). FWHM, full-width-at-half maximum. FW10%M, full-width-at-10% maximum. Scale bars, 20 μm.

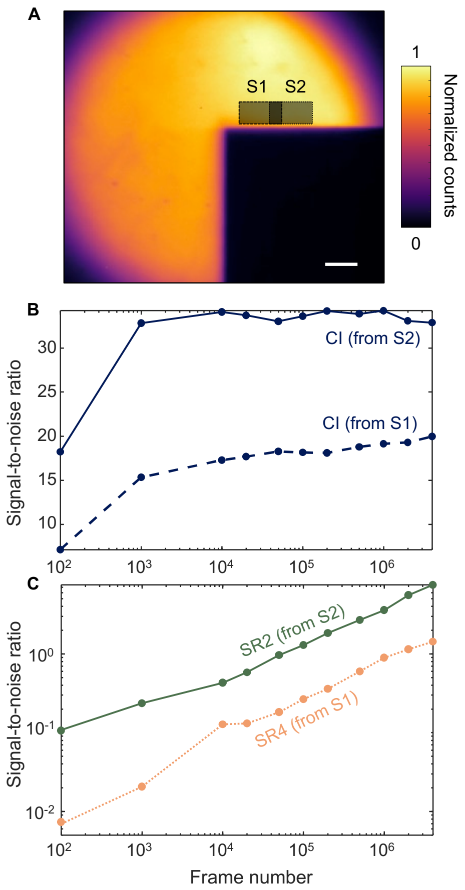

Fig. S12: **Signal-to-noise ratio (SNR) versus frame count.** (**A**) CI image of a corner of a USAF 1951 resolution target. S1 and S2 denote the regions of interest selected for signals. Both selected regions are sufficiently far away from the edge. Scale bar, 20 µm. (**B**) SNRs versus frame counts of the CI images computed using S1 (dashed line) and S2 (solid line). (**C**) SNRs versus frame counts of the SR2 images computed using S2 where the SNRs of SR2 are the greatest and the SR4 images computed using S1 where the SNRs of SR4 are the greatest.

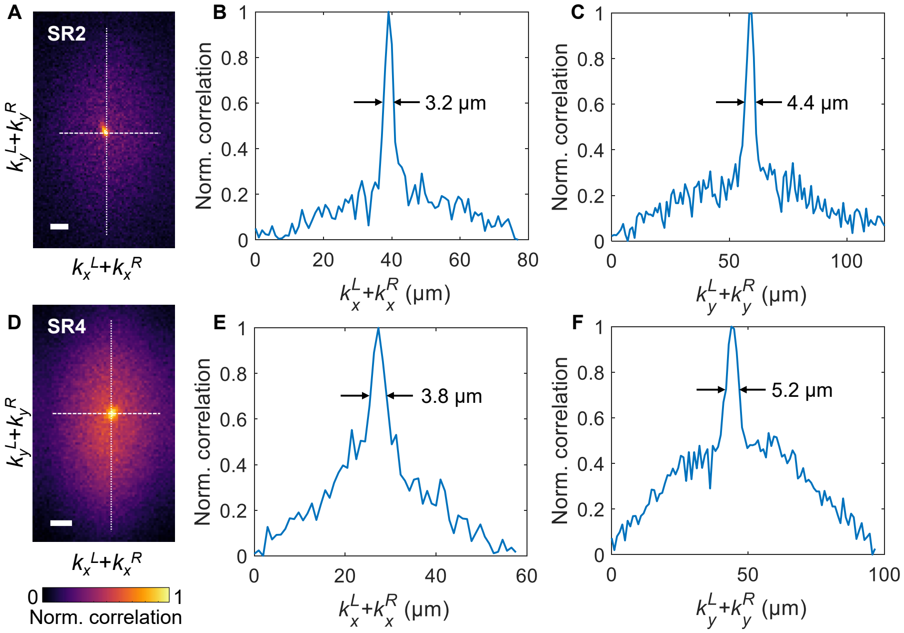

Fig. S13: **Correlation for SR2 and SR4 imaging.** (**A**) Distribution of coincidence intensity represented in the sum-coordinate axes in SR2. (**B**–**C**) Normalized intensity correlation along the (**B**) k_x and (**C**) k_y directions in SR2. (**D**) Distribution of coincidence intensity represented in the sum-coordinate axes in SR4. (**E**–**F**) Normalized intensity correlation along the (**E**) k_x and (**F**) k_y directions in SR4. Scale bars, 10 μm.

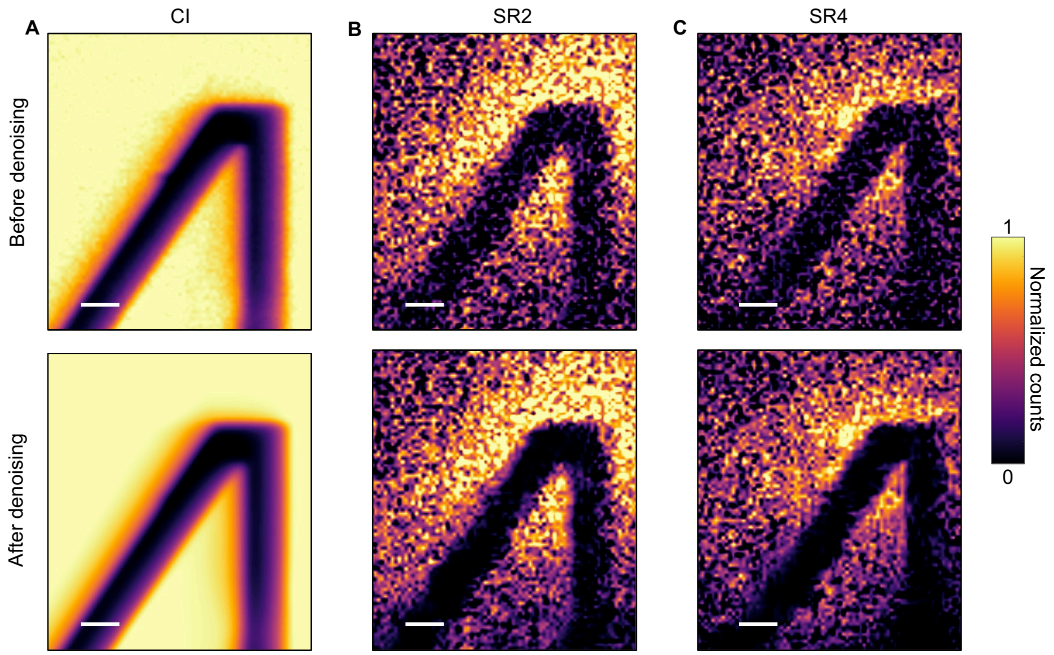

Fig. S14: **Comparison of images before and after denoising.** (**A**–**C**) Images of the number 4 in a USAF 1951 resolution target acquired using (**A**) CI, (**B**) SR2, and (**C**) SR4 before and after denoising. Scale bars, 10 µm.

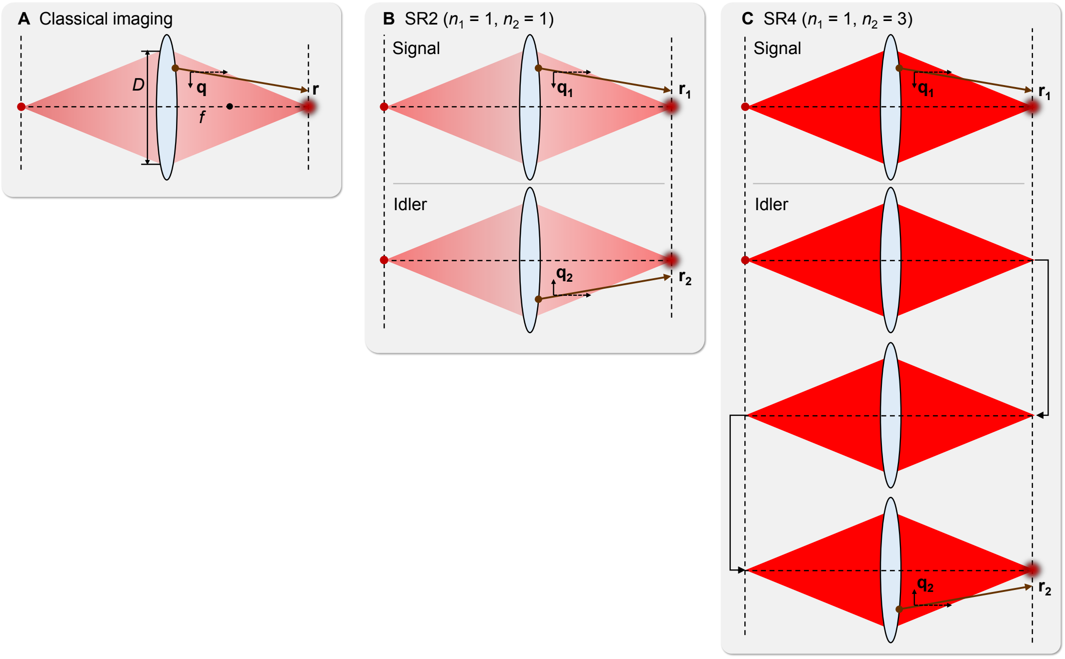

Fig. S15: **Simplified schematics of (A) classical imaging, (B) SR2, and (C) SR4.**

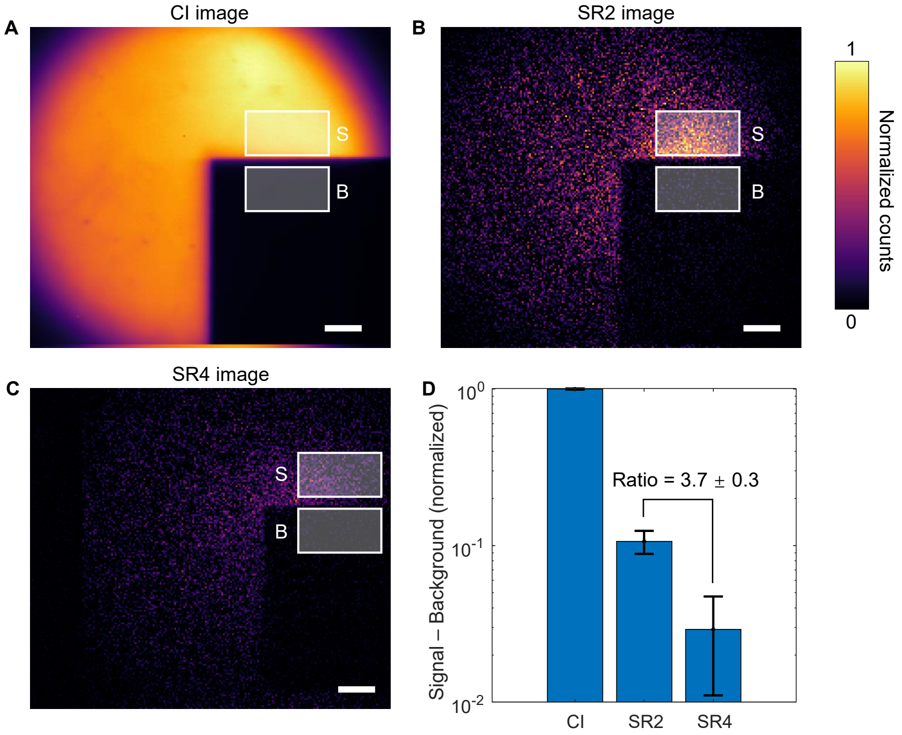

Fig. S16: **Experimental estimation of the post-selection efficiencies.** (**A**) CI image acquired in the signal arm with a USAF 1951 resolution target positioned at the object plane. (**B**, **C**) Reconstructed images obtained with SR2 (**B**) and SR4 (**C**) under identical conditions. All images are normalized to the intensity range defined by the maximum and minimum values in (**A**) to enable direct comparison. S and B indicate the selected signal and background regions, respectively. Scale bars, 10 µm. (**D**) Quantitative comparison of the mean intensity difference between S and B for CI, SR2, and SR4. Error bars denote the standard error of the mean, calculated over $n_{\mathrm{pixel}} = 800$ pixels.

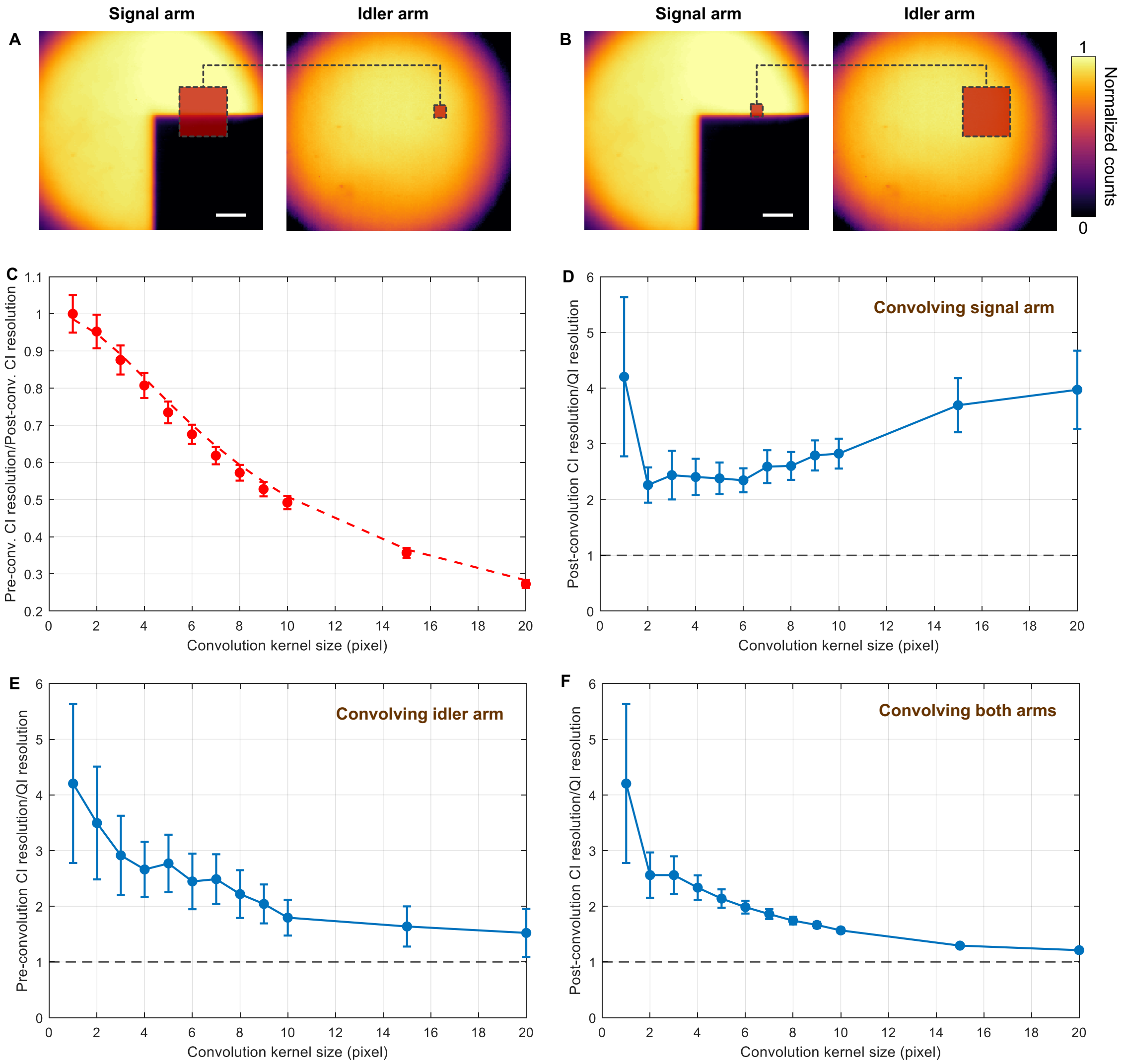

Fig. S17: **Blurring of SR4 experimental data by moving-average convolution.** (**A**–**B**) Convolution schemes. (**A**) A moving kernel of variable size is applied to the signal arm. For each idler pixel, the corresponding signal pixels within the kernel are averaged before the covariance is calculated. (**B**) A moving kernel of variable size is applied to the idler arm. For each signal pixel, the corresponding idler pixels within the kernel are averaged before the covariance is calculated. (**C**) Resolution comparison between the original and convolved signal (i.e., classical) images. Conv. denotes convolution. The dashed line indicates the theoretical prediction. (**D**) Resolution comparison between the quantum image reconstructed after convolving the signal arm and the convolved signal (i.e., classical) images. (**E**) Resolution comparison between the quantum image reconstructed after convolving the idler arm and the unconvolved signal (i.e., classical) image. (**F**) Resolution comparison between the quantum image reconstructed after convolving both arms and the convolved signal (i.e., classical) images. Each data point shows the mean ± standard error of the mean ($n = 30$). Scale bars, 10 µm.

Supplementary table

Supplementary table 1: **Numerical aperture (NA) and diffraction limit (DL) analysis of the CI setup.** The three measurements show consistent results in both NA and DL.

**Measurement**	**Full width at half maximum**		**Full width at 1/10 maximum**	
	**NA**	**DL (µm)**	**NA**	**DL (µm)**
**Between objectives (obj)** (Positions 1 and 2)	0.18	2.25	0.33	1.23
**From exit pupil (XP)** (Position 3)	0.19	2.13	0.32	1.26
**Before EMCCD (EMCCD)** (Positions 4 and 5)	0.14	2.89	0.32	1.26

REFERENCES

1. A. Lipson, S. Lipson, H. Lipson, *Optical Physics* (Cambridge Univ. Press, 2012).

2. E. Betzig, J. Trautman, Near-field optics: Microscopy, spectroscopy, and surface modification beyond the diffraction limit. *Science* **257**, 189–195 (1992).

3. X. Zhang, Z. Liu, Superlenses to overcome the diffraction limit. *Nat. Mater.* **7**, 435–441 (2008).

4. M. Minsky, Memoir on inventing the confocal scanning microscope. *Scanning* **10**, 128–138 (1988).

5. S. Hell, E. Stelzer, Properties of a 4Pi confocal fluorescence microscope. *J. Opt. Soc. Am. A* **9**, 2159–2166 (1992).

6. S. Hell, J. Wichmann, Breaking the diffraction resolution limit by stimulated emission: Stimulated-emission-depletion fluorescence microscopy. *Opt. Lett.* **19**, 780–782 (1994).

7. M. Gustafsson, Nonlinear structured-illumination microscopy: Wide-field fluorescence imaging with theoretically unlimited resolution. *Proc. Natl. Acad. Sci. U.S.A.* **102**, 13081–13086 (2005).

8. E. Betzig, G. Patterson, R. Sougrat, O. Lindwasser, S. Olenych, J. Bonifacino, M. Davidson, J. Lippincott-Schwartz, H. Hess, Imaging intracellular fluorescent proteins at nanometer resolution. *Science* **313**, 1642–1645 (2006).

9. M. Rust, M. Bates, X. Zhuang, Sub-diffraction-limit imaging by stochastic optical reconstruction microscopy (STORM). *Nat. Methods* **3**, 793–796 (2006).

10. C. Ledig, L. Theis, F. Huszár, J. Caballero, A. Cunningham, A. Acosta, A. Aitken, A. Tejani, J. Totz, Z. Wang, W. Shi, Photo-realistic single image super-resolution using a generative adversarial network, in *Proceedings of the IEEE Conference on Computer Vision and Pattern Recognition (CVPR)* (IEEE, 2017), pp. 4681–4690.

11. P.-A. Moreau, E. Toninelli, T. Gregory, M. Padgett, Imaging with quantum states of light. *Nat. Rev. Phys.* **1**, 367–380 (2019).

12. R. Horodecki, P. Horodecki, M. Horodecki, K. Horodecki, Quantum entanglement. *Rev. Mod. Phys.* **81**, 865–942 (2009).

13. V. Giovannetti, S. Lloyd, L. Maccone, Advances in quantum metrology. *Nat. Photonics* **5**, 222–229 (2011).

14. M. Taylor, W. Bowen, Quantum metrology and its application in biology. *Phys. Rep.* **615**, 1–59 (2016).

15. V. Giovannetti, S. Lloyd, L. Maccone, J. Shapiro, Sub-Rayleigh-diffraction-bound quantum imaging. *Phys. Rev. A* **79**, 013827 (2009).

16. D.-Q. Xu, X.-B. Song, H.-G. Li, D.-J. Zhang, H.-B. Wang, J. Xiong, K. Wang, Experimental observation of sub-Rayleigh quantum imaging with a two-photon entangled source. *Appl. Phys. Lett.* **106**, 171104 (2015).

17. M. Unternährer, B. Bessire, L. Gasparini, M. Perenzoni, A. Stefanov, Super-resolution quantum imaging at the Heisenberg limit. *Optica* **5**, 1150–1154 (2018).

18. M. Mitchell, J. Lundeen, A. Steinberg, Super-resolving phase measurements with a multiphoton entangled state. *Nature* **429**, 161–164 (2004).

19. P. Walther, J.-W. Pan, M. Aspelmeyer, R. Ursin, S. Gasparoni, A. Zeilinger, De Broglie wavelength of a non-local four-photon state. *Nature* **429**, 158–161 (2004).

20. M. D'Angelo, M. Chekhova, Y. Shih, Two-photon diffraction and quantum lithography. *Phys. Rev. Lett.* **87**, 013602 (2001).

21. G. Xiang, B. Higgins, D. Berry, H. Wiseman, G. Pryde, Entanglement-enhanced measurement of a completely unknown optical phase. *Nat. Photonics* **5**, 43–47 (2011).

22. C. Degen, F. Reinhard, P. Cappellaro, Quantum sensing. *Rev. Mod. Phys.* **89**, 035002 (2017).

23. M. Napolitano, M. Koschorreck, B. Dubost, N. Behbood, R. Sewell, M. W. Mitchell, Interaction-based quantum metrology showing scaling beyond the Heisenberg limit. *Nature* **471**, 486–489 (2011).

24. P. Yin, X. Zhao, Y. Yang, Y. Guo, W.-H. Zhang, G.-C. Li, Y.-J. Han, B.-H. Liu, J.-S. Xu, G. Chiribella, G. Chen, C.-F. Li, G.-C. Guo, Experimental super-Heisenberg quantum metrology with indefinite gate order. *Nat. Phys.* **19**, 1122–1127 (2023).

25. J. Beltrán, A. Luis, Breaking the Heisenberg limit with inefficient detectors. *Phys. Rev. A* **72**, 045801 (2005).

26. S. Boixo, S. Flammia, C. Caves, J. Geremia, Generalized limits for single-parameter quantum estimation. *Phys. Rev. Lett.* **98**, 090401 (2007).

27. S. Roy, S. Braunstein, Exponentially enhanced quantum metrology. *Phys. Rev. Lett.* **100**, 220501 (2008).

28. Y. Yang, Memory effects in quantum metrology. *Phys. Rev. Lett.* **123**, 110501 (2019).

29. H. Defienne, B. Ndagano, A. Lyons, D. Faccio, Polarization entanglement-enabled quantum holography. *Nat. Phys.* **17**, 591–597 (2021).

30. Z. He, Y. Zhang, X. Tong, L. Li, L. Wang, Quantum microscopy of cells at the Heisenberg limit. *Nat. Commun.* **14**, 2441 (2023).

31. T. Juffmann, B. B. Klopfer, T. L. Frankort, P. Haslinger, M. A. Kasevich, Multi-pass microscopy. *Nat. Commun.* **7**, 12858 (2016).

32. C. Wagenknecht, C.-M. Li, A. Reingruber, X.-H. Bao, A. Goebel, Y.-A. Chen, Q. Zhang, K. Chen, J.-W. Pan, Experimental demonstration of a heralded entanglement source. *Nat. Photonics* **4**, 549–552 (2010).

33. H. Zhang, X.-M. Jin, J. Yang, H.-N. Dai, S.-J. Yang, T.-M. Zhao, J. Rui, Y. He, X. Jiang, F. Yang, G.-S. Pan, Z.-S. Yuan, Y. Deng, Z.-B. Chen, X.-H. Bao, S. Chen, B. Zhao, J.-W. Pan,

Preparation and storage of frequency-uncorrelated entangled photons from cavity-enhanced spontaneous parametric downconversion. *Nat. Photonics* **5**, 628–632 (2011).

34. C. Couteau, Spontaneous parametric down-conversion. *Contemp. Phys.* **59**, 291–304 (2018).

35. P. R. Bevington, D. K. Robinson, J. M. Blair, A. J. Mallinckrodt, S. McKay, Data reduction and error analysis for the physical sciences. *Comput. Phys.* **7**, 415–416 (1993).

36. S. Daryanoosh, S. Slussarenko, D. Berry, H. Wiseman, G. Pryde, Experimental optical phase measurement approaching the exact Heisenberg limit. *Nat. Commun.* **9**, 4606 (2018).

37. L.-Z. Liu, Y.-Z. Zhang, Z.-D. Li, R. Zhang, X.-F. Yin, Y.-Y. Fei, L. Li, N.-L. Liu, F. Xu, Y.-A. Chen, J.-W. Pan, Distributed quantum phase estimation with entangled photons. *Nat. Photonics* **15**, 137–142 (2021).

38. L. V. Wang, H.-i. Wu, *Biomedical Optics: Principles And Imaging* (John Wiley & Sons, 2007).

39. L. Li, Z. Liu, X. Ren, S. Wang, V.-C. Su, M.-K. Chen, C. H. Chu, H. Y. Kuo, B. Liu, W. Zang, G. Chen, C.-F. Li, G.-C. Guo, Metalens-array–based high-dimensional and multiphoton quantum source. *Science* **368**, 1487–1490 (2020).

40. B. G. Oripov, D. S. Rampini, J. Allmaras, M. D. Shaw, S. W. Nam, B. Korzh, A. N. McCaughan, A superconducting nanowire single-photon camera with 400,000 pixels. *Nature* **622**, 730–734 (2023).

41. A. Valencia, G. Scarcelli, M. D'Angelo, Y. Shih, Two-photon imaging with thermal light. *Phys. Rev. Lett.* **94**, 063601 (2005).

42. J.-E. Oh, Y.-W. Cho, G. Scarcelli, Y.-H. Kim, Sub-Rayleigh imaging via speckle illumination. *Opt. Lett.* **38**, 682–684 (2013).

43. Z. He, Y. Zhang, X. Tong, L. Li, L. V. Wang, Heisenberg scaling quantum microscopy: Experiment and theory. arXiv:2303.04948 [quant-ph] (2025).

44. Y. Shih, Quantum imaging. *IEEE J. Sel. Top Quantum Electron.* **13**, 1016–1030 (2007).

45. K. Dabov, A. Foi, V. Katkovnik, K. Egiazarian, Image denoising by sparse 3-D transform-domain collaborative filtering. *IEEE Trans. Image Process.* **16**, 2080–2095 (2007).

46. J. W. Goodman, *Introduction to Fourier Optics* (Roberts and Company Publishers, 2005).

47. M. Born, E. Wolf, *Principles of Optics: Electromagnetic Theory of Propagation, Interference and Diffraction of Light* (Elsevier, 2013).
